# Identification of Novel Short Ragweed Pollen Allergens Using Combined Transcriptomic and Immunoproteomic Approaches

**DOI:** 10.1371/journal.pone.0136258

**Published:** 2015-08-28

**Authors:** Véronique Bordas-Le Floch, Maxime Le Mignon, Julien Bouley, Rachel Groeme, Karine Jain, Véronique Baron-Bodo, Emmanuel Nony, Laurent Mascarell, Philippe Moingeon

**Affiliations:** Stallergenes, 6 rue Alexis de Tocqueville, 92183, Antony cedex, France; French National Centre for Scientific Research, FRANCE

## Abstract

**Background:**

Allergy to short ragweed (*Ambrosia artemisiifolia*) pollen is a serious and expanding health problem in North America and Europe. Whereas only 10 short ragweed pollen allergens are officially recorded, patterns of IgE reactivity observed in ragweed allergic patients suggest that other allergens contribute to allergenicity. The objective of the present study was to identify novel allergens following extensive characterization of the transcriptome and proteome of short ragweed pollen.

**Methods:**

Following a Proteomics-Informed-by-Transcriptomics approach, a comprehensive transcriptomic data set was built up from RNA-seq analysis of short ragweed pollen. Mass spectrometry-based proteomic analyses and IgE reactivity profiling after high resolution 2D-gel electrophoresis were then combined to identify novel allergens.

**Results:**

Short ragweed pollen transcripts were assembled after deep RNA sequencing and used to inform proteomic analyses, thus leading to the identification of 573 proteins in the short ragweed pollen. Patterns of IgE reactivity of individual sera from 22 allergic patients were assessed using an aqueous short ragweed pollen extract resolved over 2D-gels. Combined with information derived from the annotated pollen proteome, those analyses revealed the presence of multiple unreported IgE reactive proteins, including new Amb a 1 and Amb a 3 isoallergens as well as 7 novel candidate allergens reacting with IgEs from 20–70% of patients. The latter encompass members of the carbonic anhydrase, enolase, galactose oxidase, GDP dissociation inhibitor, pathogenesis related-17, polygalacturonase and UDP-glucose pyrophosphorylase families.

**Conclusions:**

We extended the list of allergens identified in short ragweed pollen. These findings have implications for both diagnosis and allergen immunotherapy purposes.

## Introduction

Exposure to short ragweed (*Ambrosia artemisiifolia*) pollen is a major cause of severe type I respiratory allergy [1]. While the plant was originally native to North America, subsequent spreading to Europe makes short ragweed pollinosis a public health issue in both geographical zones, with a prevalence of IgE sensitization continuously on the rise [2,3]. A comprehensive knowledge of the allergen repertoire of this pollen is a prerequisite for accurate diagnosis and efficient immunotherapy. As of today, 10 short ragweed pollen allergens have been identified and recorded by the International Union of Immunological Societies (IUIS). This includes the pectate lyase Amb a 1, considered as the major short ragweed pollen allergen with approximately 90% of ragweed-allergic individuals exhibiting IgE reactivity to this molecule [4–6]. Several minor allergens have also been described such as Amb a 3 and Amb a 7 (two plastocyanins), Amb a 4 (a defensin homologous to Art v 1), Amb a 5 (with unknown function), Amb a 6 (a non-specific lipid transfer protein), Amb a 8 (a profilin) as well as Amb a 9 and Amb a 10 (two calcium-binding proteins) [7–10]. In addition, we recently identified as a new major allergen a cysteine protease reactive with seric IgEs from 66% of ragweed-allergic patients, recorded as Amb a 11 by the IUIS [11]. Interestingly, however, many patients display IgE reactivity toward proteins distinct from such known allergens, indicating that other allergenic components remain to be identified in short ragweed pollen [5,11].

Proteomic studies for the identification of new allergens from non-model plant species such as *A*. *artemisiifolia* are classically hindered by the paucity of protein or genomic information available in public databases. The short ragweed proteome is poorly known with only about thirty different proteins documented in public databases. To circumvent this problem, we applied a broad RNA sequencing approach to first generate a comprehensive inventory of the short ragweed pollen transcripts, then infer proteins and further characterize the proteome by mass spectrometry (MS) [12–15]. Combining this approach with IgE reactivity profiling of patients’ sera using high-resolution 2D-gel electrophoresis, we present herein a detailed characterization of the *A*. *artemisiifolia* pollen proteome and allergome, with evidence for several allergens and isoallergens.

## Methods

### Analysis and annotation of the short ragweed pollen transcriptome

Total RNAs were isolated from *A*. *artemisiifolia* pollen grains (GREER, Lenoir, NC) using the RNeasy kit (Qiagen, Courtaboeuf, France). The selection of mRNAs and the construction of a random primed library were conducted by Vertis Biotechnologies (Freising, Germany). Subsequent deep mRNA sequencing using a 454 sequencing apparatus with titanium chemistry (Roche Diagnostics, Meylan, France), *de novo* assembly with the Newbler software (Roche Diagnostics) and annotation were performed by Beckman Coulter Genomics (Grenoble, France). For each transcript, 6-frame translations were used to perform a BLASTP analysis against proteins available for the flowering plants (taxonomy ID 3398) and determine the most probable reading frames. An annotated transcriptome-derived proteome (TDP) database containing 9678 predicted protein sequences was built based upon translation(s) in frame(s) yielding a blast hit, or giving the longest translated sequences. Protein sequences shorter than 33 amino acids and without any blast hit (e-value above 10^−5^) were excluded. To identify putative short ragweed allergens by similarity to known allergens, the TPD entries were compared by BLASTP to a set of plant proteins labeled as allergens in Uniprot (“*viridiplantae* and allergen”) using the CLC Genomics Workbench 7 software (CLCbio, Aarhus, Denmark). A hit was considered as positive when the calculated e-value was smaller than 10^-5^.

A pathway analysis was performed on the assembled transcripts with the KEGG (Kyoto Encyclopedia of Genes and Genomes) Automatic Annotation Server (KAAS). KEGG orthologies (KO) were assigned using the bidirectional best hit (BBH) method and *Arabidopsis thaliana* as a gene data set.

### Analysis of the short ragweed pollen proteome

Short ragweed pollen grains were ground in liquid nitrogen, then resuspended at 1:5 (w/v) in PBS pH 7.4 (Ambion, Austin, TX) supplemented with a cocktail of protease inhibitors (Complete, Roche, Meylan, France). After gentle shaking at room temperature for 1 hour and centrifugation at 10,000 *g* for 30 min, supernatants were collected, filtered at 0.22 μm, and enriched in low abundance proteins using the Proteominer kit (Bio-Rad, Marne La Coquette, France). Proteins were digested with trypsin prior to analysis by reversed-phase liquid chromatography using an Ultimate 3000 RS nano LC system (Thermo Fisher Scientific, Villebon sur Yvette, France) coupled to MS (Impact HD, Bruker Daltonics, Wissembourg, France). Peptide identification was performed using the PEAKS software (Bioinformatics Solutions Inc., Waterloo, Canada) and the in-house TDP database supplemented with Amb a 4, Amb a 5, Amb a 6 sequences obtained from IUIS (www.allergen.org) as they were not retrieved through our transcriptome analysis. Only proteins identified with a minimum of 2 peptide sequences, including at least 1 unique sequence, were taken into account. Further details on MS analysis and protein identification are provided in the online repository (In-solution MS analyses).

To create a proteome map, proteins from an aqueous short ragweed pollen extract were first precipitated using the PerfectFocus kit (Agro-Bio, La Ferté Saint Aubin, France), resuspended in a 7 M urea, 2 M thiourea, 4% CHAPS and 30 mM Tris pH 8.8 buffer, before 2D-gel electrophoresis using 3–10 non linear pH range 12.5% DALT gels (GE Healthcare, Velizy-Villacoublay, France), as per the manufacturers’ instructions. Following Sypro Ruby staining (Life Technologies, Saint Aubin, France), protein spots of interest were excised from 2D-gels using an EXquest spot cutter (Bio-Rad), then submitted to tryptic digestion and analyzed by LC-MS/MS. Further details on MS analyses and protein identification are provided in the online repository (In-gel MS analyses).

### Identification of IgE reactive proteins in short ragweed pollen

Sera from 22 European patients allergic to short ragweed pollen, enrolled in a phase I study (ClinicalTrials.gov identifier: NCT01224834) after approval by a local ethical committee, Medical Research Council Ethics Committee for Clinical Pharmacology (Hungary; EudraCT: 2008-003715-12), were used to detect short ragweed pollen proteins exhibiting IgE reactivity. Written informed consents were obtained from patients. All patients had a clinical history of seasonal ragweed pollen allergy, a positive skin prick test and specific IgE levels to short ragweed pollen allergens ≥ 0.70 kU/L documented by ImmunoCap (Thermo Fisher Scientific, Saint Quentin en Yvelines, France). For immunoblotting, total proteins from an aqueous short ragweed pollen extract were fractionated by 2D-PAGE as described above, blotted onto a nitrocellulose membrane, incubated with individual sera at a 1:10 dilution, and then with rabbit anti-human IgE (Dako, Les Ulis, France) followed by HRP-conjugated goat anti-rabbit antibodies (Merck Millipore, Molsheim, France). Specific IgE binding was detected by chemiluminescence using the Advansta WesternBright Quantum kit (Diagomics, Blagnac, France) and a Fusion FX CCD camera (Vilber Lourmat, Collegien, France). IgE reactive spots were annotated using the proteome map.

## Results

### Short ragweed pollen transcriptome and proteome analyses

**Fig 1:**
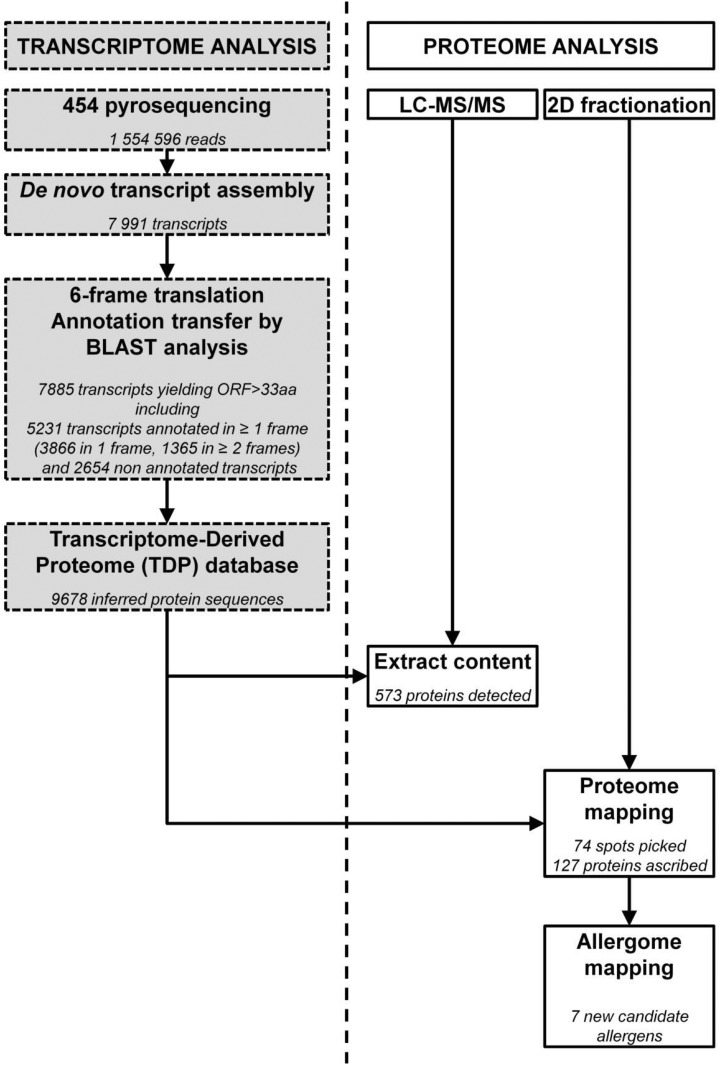
Experimental workflow of short ragweed pollen transcriptome, proteome and allergome characterization. Messenger RNAs from short ragweed pollen were analyzed by deep RNA sequencing using 454 sequencing technology. After de novo transcript assembly, a translated sequence database was generated and used to assign MS/MS spectra from proteomics experiments and perform homology searches. After obtaining a reference 2D-map of the short ragweed pollen proteome, IgE reactivity was analyzed with sera from 22 allergic patients.

**Fig 2:**
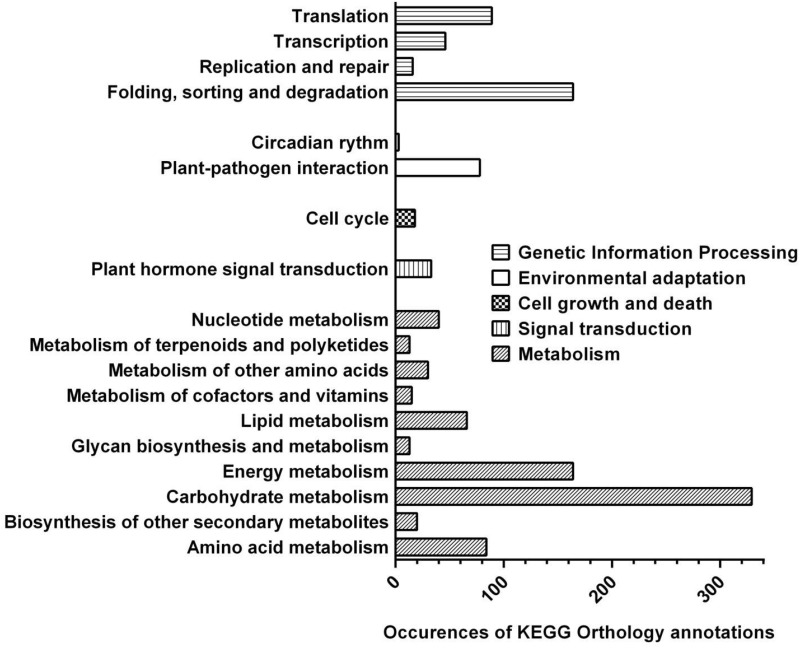
Functional analysis of the short ragweed pollen proteome. Proteome mining and classification into functional categories was performed using the KAAS server. The histogram denotes numbers of occurrences of KEGG Orthology (KO) annotations (abscissa) assembled in selected categories (ordinate). Among allergens, Amb a 1 and carbonic anhydrase were related to carbohydrate and energy metabolisms, respectively.

To characterize the short ragweed pollen proteome and search for novel allergens, a combined approach relying on deep RNA sequencing, MS analyses and IgE reactivity mapping was applied (Fig 1). As a first step, mRNAs were extracted from short ragweed pollen and sequenced using 454 Next Generation Sequencing technology. A total of 1 554 596 raw reads, with a 20-580 bp (median 468 bp) length distribution, was obtained. The short ragweed pollen transcriptome was then rebuilt by assembling the sequencing reads, thus yielding 7991 transcripts. Protein sequences derived from such transcripts were compiled in a Transcriptome-Derived Proteome (TDP) database compatible with MS search engines. Up to 7885 transcripts gave rise by translation to 9678 peptides sequences at least 33 amino acid long, out of which 5231 exhibited significant homology to recorded plant proteins in at least one translation frame. To further complement the annotation of this library, a pathway mapping analysis was performed using the KEGG Automatic Annotation Server (KAAS). As expected, such annotation revealed many transcripts related to molecules involved in either primary metabolic pathways (*e*. *g*. carbohydrate, amino acid, nucleotide or energy metabolisms), genetic information processing, plant-pathogen interaction, cell growth and death or signal transduction (Fig 2). In addition, some transcripts pertaining to specialized metabolisms (*e*. *g*. terpenoid, phenylpropanoid synthesis) were also found.

We then analyzed a short ragweed pollen extract by LC-MS/MS, prior and after protein enrichment to assess low abundance proteins and improve the proteome coverage. A total of 573 protein groups were detected, based on at least 2 peptides identified, out of which ≥ 1 unique (S1 Table).

### Short ragweed pollen allergome analyses

Focusing specifically on allergens, our LC-MS/MS analysis confirmed, using the aforementioned criteria, the presence in the short ragweed pollen extract of both Amb a 1 (5 known isoforms, as well as 4 other Amb a 1-related proteins), Amb a 5, Amb a 6, Amb a 8, Amb a 10 and Amb a 11. Although the registered Amb a 3 allergen was not retrieved by our analyses, a highly similar protein displaying 87% overall sequence identity (and up to 96.7% identity within the 91 amino-terminal residues) with Amb a 3 was identified (S1 Table). In addition, we also detected proteins exhibiting sequence similarities with known plant allergens of the enolase, expansin, isoflavone reductase, Ole e 1, polygalacturonase and pathogenesis-related (PR) 2 families.

**Fig 3:**
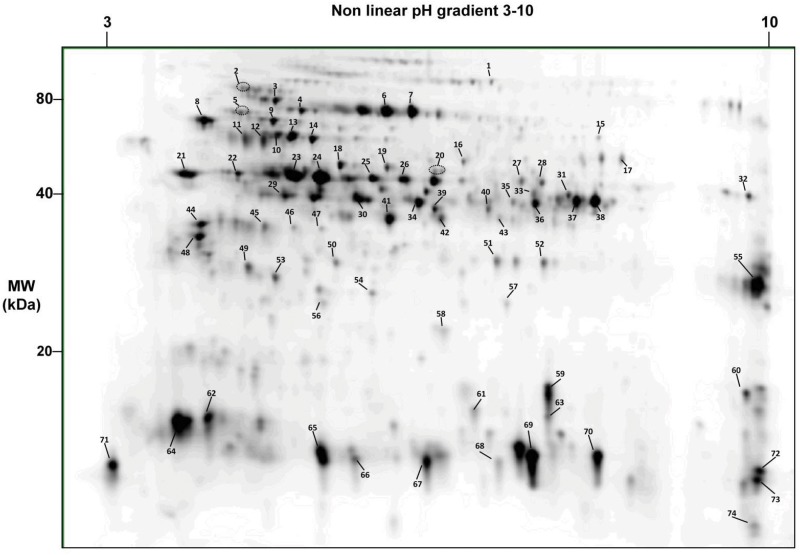
2D-gel reference map of the short ragweed pollen proteome. Proteins from an aqueous short ragweed pollen extract were separated by 2D-gel electrophoresis and stained with Sypro Ruby. Proteins spots were picked and analyzed by LC-MS/MS after trypsin digestion. Proteins were identified using the Transcriptome-Derived Proteome collection supplemented with missing known allergens. Numbers refer to spots analyzed by mass spectrometry. Identification details are provided in Table 1.

**Fig 4:**
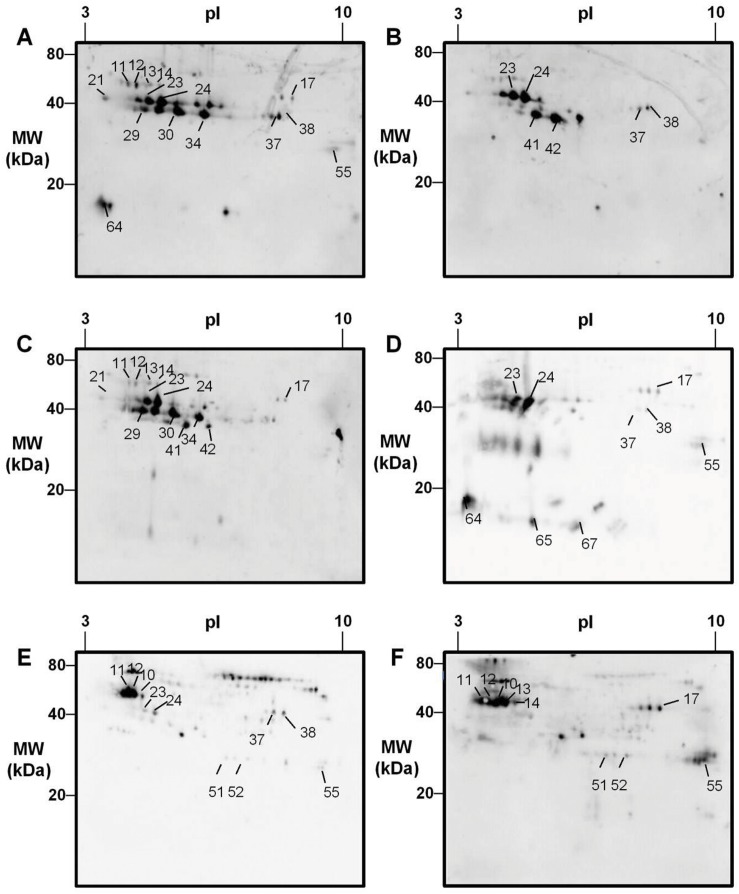
Mapping of IgE reactivity with short ragweed pollen proteins. Water soluble short ragweed pollen proteins were resolved by 2D-gel electrophoresis, then probed by western blot with seric IgEs from 22 ragweed-allergic patients. Representative patterns of IgE reactivity obtained from 6 patients (a-f) are shown. Identification details are provided in Table 1.

To complement those analyses, we fractionated a short ragweed extract using high-resolution 2D-PAGE (Fig 3). Gel plugs from 74 protein spots were recovered, trypsin digested and analyzed by LC-MS/MS. A total of 127 protein entries were identified in these spots (with 2 or more peptides sequenced, including at least 1 unique, data not shown). We subsequently assessed IgE reactivity of short ragweed pollen proteins by immunoblotting with individual sera from 22 allergic individuals. As shown in Fig 4a–4f, most patients had seric IgEs reactive with multiple ragweed proteins. Up to 49 IgE reactive spots, out of which 25 exhibiting IgE reactivity with ≥ 20% of sera tested, were assigned using the 2D proteome map (Table 1).

**Table 1:** Assignments of 2D-gel spots containing IgE reactive proteins. An aqueous short ragweed pollen extract was submitted to 2D-gel electrophoresis followed by western blotting on nitrocellulose membranes with seric IgEs from 22 ragweed-allergic patients. A total of 74 gel plugs (with spot numbers referring to Fig 3) were recovered and assigned after LC-MS/MS analysis using the TDP database. For each spot analyzed, the top 3 master proteins identified in each protein group, based on ≥ 2 peptides sequenced, including ≥ 1 unique one, are listed by decreasing occurences of peptide matches as well as prevalences of IgE reactivity among patients. When several proteins shared the same annotation, a ranking number (#) was assigned.

Spot numbers	IgE reactivity frequencies	Identification	Total (unique) number of peptides sequenced
1	<20%	Copper binding protein #1	2 (2)
2	<20%	Galactose oxidase #1	10 (10)
		Amb a 1.01	2 (2)
3	<20%	Galactose oxidase #1	24 (24)
		Galactose oxidase #2	2 (2)
4	-	Galactose oxidase #1	8 (8)
		Phosphoglycerate mutase	3 (3)
		Galactose oxidase #2	3 (3)
5	<20%	Malic enzyme	21 (21)
		Rab GDP-dissociation inhibitor #1	7 (6)
		Polygalacturonase #1	6 (6)
6	31%	Galactose oxidase #2	26 (26)
		Phosphoglucomutase	13 (13)
		Berberine bridge enzyme	12 (12)
7	31%	Galactose oxidase #2	28 (28)
		Phosphoglucomutase	13 (13)
		Berberine bridge enzyme	7 (7)
8	32%	Rab-GDP dissociation inhibitor #2	26 (23)
		Amb a 1.01	4 (4)
		Rab GDP-dissociation inhibitor #1	4 (1)
9	27%	Rab GDP-dissociation inhibitor #1	20 (18)
		Rab-GDP dissociation inhibitor #2	8 (6)
		UDP-glucose pyrophosphorylase	6 (6)
10	41%	UDP-glucose pyrophosphorylase	18 (18)
		Polygalacturonase #1	6 (6)
		Enolase	4 (4)
11	68%	UDP-glucose pyrophosphorylase	20 (20)
		Polygalacturonase #1	16 (16)
		ATP synthase subunit beta	12 (12)
12	68%	UDP-glucose pyrophosphorylase	25 (25)
		Polygalacturonase #1	17 (17)
		ATP synthase subunit beta	14 (14)
13	50%	UDP-glucose pyrophosphorylase	34 (34)
		Polygalacturonase #1	9 (9)
		Enolase	9 (7)
14	50%	UDP-glucose pyrophosphorylase	36 (36)
		Polygalacturonase #1	6 (6)
		Enolase	5 (4)
		Amb a 1.03	5 (2)
15	<20%	Fructose-bisphosphate aldolase #1	26 (26)
		Pectinesterase	11 (11)
		Amb a 1-like #1	10 (7)
16	-	UDP-glucose pyrophosphorylase	39 (39)
		Enolase	7 (6)
		Polygalacturonase #1	6 (6)
		Amb a 1.04	6 (5)
17	45%	Polygalacturonase #2	16 (16)
		Amb a 1.02	11 (11)
		Galactose oxidase #2	8 (8)
18	-	Phosphoglycerate kinase	10 (10)
		Amb a 1.01	9 (8)
		Amb a 1.05	7 (6)
19	-	UDP-glucose pyrophosphorylase	26 (26)
		Phosphoglycerate kinase	11 (11)
		Enolase	6 (6)
		Polygalacturonase #1	6 (6)
20	<20%	Isocitrate dehydrogenase	7 (7)
		Amb a 1.05	5 (5)
		Phosphoglycerate kinase	5 (5)
21	41%	Amb a 1-like #2	21 (20)
		Amb a 1-like #3	5 (3)
		Pantothenate kinase	3 (3)
		UDP-glucose pyrophosphorylase	3 (3)
		14-3-3-like protein #1	3 (1)
22	<20%	Isocitrate dehydrogenase	16 (16)
		Hexokinase	2 (2)
		UDP-glucose pyrophosphorylase	2 (2)
		Amb a 1.05	2 (2)
23	95%	Amb a 1.01	27 (21)
		Amb a 1.03	13 (9)
		Amb a 1.04	12 (8)
24	95%	Amb a 1.01	32 (27)
		Amb a 1.03	14 (11)
		Amb a 1.04	9 (5)
25	<20%	Amb a 1.05	18 (15)
		Amb a 1.03	12 (10)
		Amb a 1.01	9 (8)
26	<20%	Amb a 1.05	22 (19)
		Amb a 1.03	5 (3)
		Fructose-bisphosphate aldolase #2	4 (4)
27	<20%	Amb a 1-like #1	11 (11)
		Alcohol dehydrogenase #1	9 (9)
		Amb a 1.02	9 (8)
28	<20%	Alcohol dehydrogenase #1	14 (14)
		Amb a 1-like #1	13 (13)
		Amb a 1.02	13 (12)
		Pectinesterase	5 (5)
		Alcohol dehydrogenase #2	4 (4)
		Glyceraldehyde-3-phosphate dehydrogenase #1	3 (3)
29	95%	Amb a 1.04	16 (13)
		Amb a 1.03	12 (9)
		Amb a 11	4 (4)
		Fructose-bisphosphate aldolase #2	4 (4)
		Amb a 1.01	4 (2)
		Amb a 1.02	4 (1)
30	95%	Amb a 1.03	19 (19)
		Fructose-bisphosphate aldolase #2	14 (14)
		Amb a 1.04	6 (5)
31	<20%	Amb a 1-like #1	22 (16)
		Amb a 1.02	18 (15)
		Fructose-bisphosphate aldolase #2	10 (10)
		Glyceraldehyde-3-phosphate dehydrogenase #2	8 (2)
		Glyceraldehyde-3-phosphate dehydrogenase #3	8 (2)
32	<20%	Amb a 1-like #4	16 (16)
33	<20%	Amb a 1-like #1	21 (15)
		Amb a 1.02	14 (10)
		Fructose-bisphosphate aldolase #2	13 (13)
34	95%	Amb a 1.03	22 (21)
		Fructose-bisphosphate aldolase #2	14 (14)
		Type IIIa membrane protein cp-wap13	6 (6)
35	-	Fructose-bisphosphate aldolase #2	24 (24)
		Amb a 1.02	12 (9)
		Glyceraldehyde-3-phosphate dehydrogenase #4	8 (2)
36	<20%	Pectinesterase	11 (11)
		Amb a 1.02	11 (9)
		Fructose-bisphosphate aldolase #2	10 (10)
37	73%	Amb a 1.02	24 (20)
		Amb a 1-like #1	12 (12)
		Pectinesterase	11 (11)
38	73%	Amb a 1.0202	30 (27)
		Glyceraldehyde-3-phosphate dehydrogenase #1	7 (5)
		Amb a 1-like #1	5 (5)
		Fructose-bisphosphate aldolase #2	5 (5)
39	-	Fructose-bisphosphate aldolase #2	12 (12)
		Amb a 11	5 (5)
		Aldose 1-epimerase family protein	5 (5)
		Amb a 1.01	5 (3)
		Glyceraldehyde-3-phosphate dehydrogenase #4	5 (3)
40	<20%	Amb a 1-like #3	19 (11)
		Amb a 1.04	14 (10)
		Amb a 1.03	10 (6)
41	64%	Amb a 11	12 (12)
		Transducin	8 (8)
		Fructose-bisphosphate aldolase #2	7 (7)
42	64%	Amb a 11	11 (11)
		Fructose-bisphosphate aldolase #2	11 (11)
		Aldose 1-epimerase family protein	8 (8)
43	-	Fructose-bisphosphate aldolase #1	29 (29)
		Aldose 1-epimerase family protein	9 (9)
		Phenylcoumaran benzylic ether reductase-like protein	6 (6)
44	<20%	14-3-3 protein #1	10 (7)
		14-3-3 protein #2	10 (7)
		14-3-3 protein #3	6 (6)
45	-	Cysteine protease	4 (4)
		Lactoylglutathione lyase	4 (4)
		Actin	3 (1)
		Actin-97	3 (1)
46	-	Lactoylglutathione lyase	3 (3)
47	-	Lactoylglutathione lyase	12 (12)
		Fructose-bisphosphate aldolase #2	10 (10)
		Pseudouridine-metabolizing bifunctional protein #1	10 (1)
48	-	14-3-3 protein #2	10 (8)
		14-3-3 protein #3	8 (8)
		14-3-3 protein #1	8 (6)
49	<20%	Amb a 1.01	4 (4)
		14-3-3 protein #3	4 (4)
		Triosephosphate isomerase	4 (4)
50	-	Pseudouridine-metabolizing bifunctional protein #1	18 (18)
		Carboxylesterase	9 (9)
		Ascorbate peroxidase	8 (8)
51	27%	A-type carbonic anhydrase	15 (15)
		Fructose-bisphosphate aldolase #2	2 (2)
52	27%	A-type carbonic anhydrase	14 (14)
		Villin	3 (3)
		Copper binding protein #2	2 (2)
		Glucose and ribitol dehydrogenase	2 (2)
53	<20%	Triosephosphate isomerase	6 (6)
		Aldose 1-epimerase family protein	5 (5)
		Amb a 1.02	5 (4)
54	-	Amb a 1.02	4 (2)
		Actin	3 (3)
		Glutathione S-transferase	3 (3)
55	36%	Pathogenesis-related protein 17	4 (4)
56	-	Amb a 1.02	10 (8)
		14-3-3 protein #3	7 (7)
		Inorganic pyrophosphatase	7 (7)
57	-	Glutathione S-transferase	8 (8)
		Amb a 1-like #1	6 (5)
		Amb a 1-like #4	3 (2)
		Actin	3 (1)
		Actin-97	3 (1)
58	-	Art v 2 allergen	12 (12)
		Amb a 11	5 (5)
		Actin	5 (2)
59	<20%	Amb a 3	10 (10)
		Copper binding protein #2	3 (3)
60	-	UDP-glucose pyrophosphorylase	5 (5)
		Actin	2 (2)
		Enolase	2 (2)
		Phosphoglycerate kinase	2 (2)
61	55%	Amb a 1.01	15 (10)
		Amb a 1.04	12 (6)
		Amb a 1-like #1	7 (7)
62	-	Cystatin proteinase inhibitor	6 (6)
		UDP-glucose pyrophosphorylase	5 (5)
		Thioredoxin	3 (3)
		Actin #2	3 (3)
		Copper binding protein # 6	3 (3)
63	-	Amb a 3-like	12 (12)
		Villin	3 (3)
		Pseudouridine-metabolizing bifunctional protein #2	3 (3)
		Copper binding protein #3	3 (3)
		Actin #3	3 (1)
		Actin 4 #2	3 (1)
64	50%	Copper binding protein #2	5 (5)
		Amb a 8.0102	4 (3)
		Amb a 8.0101	4 (3)
65	<20%	Copper binding protein #4	3 (3)
		Copper binding protein #1	2 (2)
66	-	Copper binding protein #4	3 (3)
		Copper binding protein #1	2 (2)
67	<20%	Copper binding protein #3	5 (3)
		Copper binding protein #4	4 (2)
		Copper binding protein #5	2 (2)
68	<20%	Copper binding protein #4	12 (7)
		Copper binding protein #2	9 (4)
		Glutaredoxin	3 (3)
		Amb a 1.01	3 (2)
		Copper binding protein #5	3 (1)
69	<20%	Copper binding protein #2	13 (9)
		Copper binding protein #4	7 (3)
		Glutaredoxin	6 (6)
70	-	Copper binding protein #2	9 (9)
		Glutaredoxin C4	5 (5)
		Ras-related protein	3 (3)
71	-	Calmodulin	12 (12)
		Villin #2	5 (5)
		Actin 4	4 (3)
72	-	Copper binding protein #2	3 (3)
		Early nodulin 55–2 putative	3 (3)
73	-	Early nodulin 55–2 putative	3 (3)
		Copper binding protein #2	3 (3)
74	-	Amb a 5.0101	2 (2)

As expected from the known high abundance of its various isoallergens in short ragweed pollen, Amb a 1 and fragments thereof were identified in several spots. Amb a 1 was confirmed as the most prevalent major allergen, with 95.5% (21/22) of patients’ sera reacting with at least one Amb a 1 spot (Fig 4, spots 23, 24, 29, 30, 34 and 37). In addition, a new acidic Amb a 1-like molecule (S2 Table) was identified in spot 21, reactive with seric IgEs from 41% (9/22) of patients. Spots 27, 31–33 and 40 also contained Amb a 1-related proteins, albeit surprisingly with no or marginal IgE reactivity. Spots 41, 42, containing the recently described Amb a 11 major allergen, reacted with IgEs from 63.5% (14/22) of patients. Amb a 8 (spot 64) as well as the Amb a 3-like molecule (spot 59) were recognized by IgEs from 50% (11/22) and 18% (4/22) of patients, respectively. In contrast, no IgE reactive spots were ascribed to Amb a 4, Amb a 5, Amb a 6, Amb a 9 and Amb a 10 allergens in our analyses.

We subsequently focused on groups of spots unrelated to known short ragweed pollen allergens, as they exhibited intermediate to high (20–70%) prevalences of IgE reactivity (Fig 4; Table 1; S2 Table). For example, the acidic string of spots 10-14 reacted with 41–68% (9-15/22) of patients’ sera. MS/MS analyses indicated that these spots contain proteins belonging to either the enolase, polygalacturonase or UDP-glucose pyrophosphorylase families. Another polygalacturonase with a more alkaline isoelectric point was identified in spot 17, exhibiting IgE reactivity with 45.5% (10/22) of sera. These two polygalacturonases display 68% sequence identity, and thus could be considered as isoallergens. Spot 55, recognized by IgEs from 36% (8/22) of patients, contains a homolog of the PR-17 PRp27 tobacco protein. Three additional series of spots reacted with IgE from 20–30% of patients. Peptides derived from these spots matched with members of either the carbonic anhydrase, galactose oxidase or GDP dissociation inhibitor families (Fig 4; Table 1; spots 6–7, 8–9, 51–52, respectively). Beside those 7 proteins, we also identified several plastocyanin-like copper binding proteins displaying reactivity with about 10–20% of patients’ sera (Table 1; spots 65, 67–69).

## Discussion

The pollen of *A*. *artemisiifolia* (short ragwed) causes severe allergic rhinoconjunctivitis [8]. While the rate of IgE sensitization to ragweed is about 25% in the US population [1], short ragweed spreading leads to a growing prevalence of sensitization in Europe as well [3]. Among the 10 short ragweed pollen allergens officially recognized by the IUIS as of today, the pectate lyase Amb a 1 and the cysteine protease Amb a 11 represent major allergens [8,11], whereas other allergens are considered as minor [7,8,10]. In addition however, many patients exhibit significant IgE reactivity to proteins beyond those known allergens, justifying the search for novel ragweed allergens potentially important for diagnosis and immunotherapy purposes [5,11].

To further document the *A*. *artemisiifolia* pollen proteome and allergome, we implemented a Proteomics-Informed-by-Transcriptomics approach [14] relying upon transcriptome sequencing combined with a comprehensive proteomic analysis. Data obtained with this approach were cross-tabulated with IgE reactivity profiles from sera of 22 ragweed allergic patients in order to identify novel allergens. Up to 7991 assembled transcript sequences were used to create a database comprising 9678 derived protein sequences annotated by sequence similarity with protein records from public databases. Up to 66% of those transcripts yielded annotated protein sequences in at least one reading frame, thus arguing for the good accuracy of sequence prediction in those analyses. The latter was further confirmed by the high number (n = 328) of protein groups identified by MS, with good coverage (*i*. *e*. ≥ 5 peptides sequenced; S1 Table). As expected, mining of the short ragweed pollen trancriptome confirmed the presence of many primary metabolism-related transcripts, as previously described in the plant kingdom [16,17]. In addition, specialized metabolism-related transcripts encoding proteins involved in terpenoid or phenylpropanoid synthesis were also found, in agreement with previous studies on the short ragweed transcriptome [18,19].

Many of the known short ragweed pollen allergens, such as Amb a 1, Amb a 8, Amb a 9, Amb a 10 and Amb a 11, were retrieved by the transcriptomic analysis, even if not all isoforms described for those molecules were detected. The latter could reflect either natural polymorphisms, variations in the expression of isoforms or aggregation of highly related transcripts under consensus sequences during the assembly of sequencing reads. Sequencing read mappings highlighted recurrent nucleotide point variations, confirming the existence of isoforms/variants for many allergens. As an illustration, multiple potential Amb a 1-like sequences were identified based on our transcriptome analysis, with three of them further confirmed at the proteomic level with good sequence coverage. In contrast, the presence of a single Amb a 11 transcript in our data set, without any closely related sequence detected, suggests that this major allergen exhibits limited polymorphism. Interestingly, whereas we failed to detect the Amb a 3 allergen both in transcriptomic and proteomic analyses, a highly similar molecule was identified, with 96.7% amino acid sequence identity over the amino terminal part, but with a different C-terminal end, consistent with results from a recent study on the influence of ozone exposure on the short ragweed pollen transcriptome [18].

Immunoblotting experiments confirmed IgE reactivities for Amb a 1, Amb a 8 and Amb a 11 in agreement with the literature [8,9,11,20]. We also confirmed distinct levels of IgE reactivity among Amb a 1 isoallergens, with Amb a 1.01 and Amb a 1.03 being the most frequently recognized isoallergens, as previously suggested by others [20–22]. The aforementioned novel Amb a 3-like protein displays a significantly lower IgE reactivity when compared with the known Amb a 3 allergen [8,9,23], possibly due to either an influence of the divergent C-terminal 11 amino acids or a potential denaturation of the molecule during 2D-electrophoresis. Despite the fact that they were clearly detected in the extract, we could not identify minor allergens such as Amb a 4, Amb a 5, Amb a 6, Amb a 9 and Amb a 10 [5–8,10,23] within IgE reactive spots. Only two Amb a 4 (spots 49, 53) and Amb a 5 (spot 74; Table 1)-derived peptides were detected in low or unreactive spots, in agreement with Asero et al. who also failed to detect Amb a 4 in 2D immunoblots [24]. The reason for this discrepancy in the detection of selected minor allergens is presently unclear, but could be due to limited solubility, alkaline pI, poor focusing, low abundance or epitope loss affecting IgE reactivity with those molecules. Noteworthy, Amb a 4, Amb a 5 and Amb a 6 were also undetected during the transcriptome analysis, suggesting a low mRNA abundance, as previously reported by others for Amb a 5 [25]. Alternatively, the expression of genes encoding some of those allergens could be significantly modulated during plant development. In this regard, it is interesting to note that Amb a 4 and Amb 6 were initially cloned from ragweed flowers as opposed to pollen [10,26].

Importantly, we identified at least 7 new water-soluble IgE reactive pollen proteins. Two of them, *i*. *e*. the galactose oxidase and PR-17 proteins, were detected in IgE reactive spots without other contaminating proteins (spots 3 and 55). Although they do not display homologies with known allergens, these molecules are nevertheless most probably *bona fide* allergens. Interestingly, the PR-17 protein belongs to the pathogenesis-related protein family encompassing numerous plant allergens, even if no member of class 17 had been previously described as an allergen [27–29]. Noteworthy, the 5 additional candidate allergens, namely carbonic anhydrase, enolase, GDP dissociation inhibitor, polygalacturonase and UDP-glucose pyrophosphorylase, belong to allergen families previously reported in other natural sources, including plants [30–33]. Among those, the enolase and polygalacturonase exhibit a high prevalence (41–68%) of IgE reactivity in ragweed-allergic patients, in line with the documented allergenicity of those protein families [31] Our findings are also in agreement with a recent study on the effect of NO_2_ on *A*. *artemisiifolia* pollen allergenicity concluding that enolase is a potential allergen [34]. Furthermore, the strong IgE reactivity observed for those candidate allergens in 4 of the 22 patients tested (Fig 4e and 4f), suggests that they may significantly contribute to allergy symptoms in selected patients. Lastly, additional molecules previously identified as allergens in other natural plant sources (*e*. *g*. several copper binding proteins, a PR-2 member, an expansin, an isoflavone reductase) [31] were also detected in the short ragweed pollen, even if we observed little or no IgE binding for those molecules.

Altogether, our study demonstrates, in line with parallel reports [12–15,35], that RNA sequencing coupled to MS-based proteomics and IgE-reactivity profiling is a powerful approach for the comprehensive characterization of allergens derived from natural sources. Using this strategy, we significantly expanded our knowledge of the short ragweed pollen allergome, as well as our understanding of the diversity of IgE sensitization among patients. Whereas the prevalence of IgE reactivity and clinical relevance of these candidate allergens should be further addressed in dedicated studies relying upon purified natural or properly folded recombinant molecules, we conclude that other allergens beyond the ones currently registered should be taken into account for the diagnosis and immunotherapy of short ragweed pollen allergy.

## Supporting Information

S1 File Supplementary materials. (DOCX)

S1 Table Overview of the short ragweed pollen proteome. A short ragweed pollen extract was analyzed by LC-MS/MS (prior and after protein abundance normalization). Protein identification was performed using the PEAKS software and the annotated TDP database. Only the master proteins in each protein group are reported. The transcript number, total number of peptides and number of unique peptides sequenced are provided as well as, when available, the Uniprot entry and annotation of a known homolog.
(PDF)

S2 Table Nucleotide sequences of candidate ragweed pollen allergens. S2 Table provides the nucleotide sequences, recovered from the transcriptome analysis, encoding the identified candidate allergens.
(PDF)

## References

[1] Arbes SJ Jr, Gergen PJ, Elliott L, Zeldin DC (2005) Prevalences of positive skin test responses to 10 common allergens in the US population: results from the third National Health and Nutrition Examination Survey. J Allergy Clin Immunol 116: 377–383. 16083793 10.1016/j.jaci.2005.05.017

[2] D'Amato G, Cecchi L, Bonini S, Nunes C, Annesi-Maesano I, Behrendt H, et al (2007) Allergenic pollen and pollen allergy in Europe. Allergy 62: 976–990. 17521313 10.1111/j.1398-9995.2007.01393.x

[3] Burbach GJ, Heinzerling LM, Rohnelt C, Bergmann KC, Behrendt H, Zuberbier T (2009) Ragweed sensitization in Europe—GA(2)LEN study suggests increasing prevalence. Allergy 64: 664–665. 10.1111/j.1398-9995.2009.01975.x 19210367

[4] Jahn-Schmid B, Hauser M, Wopfner N, Briza P, Berger UE, Asero R, et al (2012) Humoral and cellular cross-reactivity between Amb a 1, the major ragweed pollen allergen, and its mugwort homolog Art v 6. J Immunol 188: 1559–1567. 10.4049/jimmunol.1102445 22205029

[5] Asero R, Wopfner N, Gruber P, Gadermaier G, Ferreira F (2006) Artemisia and Ambrosia hypersensitivity: co-sensitization or co-recognition? Clin Exp Allergy 36 658–665. 16650052 10.1111/j.1365-2222.2006.02477.x

[6] Gadermaier G, Wopfner N, Wallner M, Egger M, Didierlaurent A, Regl G, et al (2008) Array-based profiling of ragweed and mugwort pollen allergens. Allergy 63: 1543–1549. 10.1111/j.1398-9995.2008.01780.x 18925891

[7] Wopfner N, Gruber P, Wallner M, Briza P, Ebner C, Mari A, et al (2008) Molecular and immunological characterization of novel weed pollen pan-allergens. Allergy 63: 872–881. 10.1111/j.1398-9995.2008.01635.x 18588553

[8] Wopfner N, Gadermaier G, Egger M, Asero R, Ebner C, Jahn-Schmid B, et al (2005) The spectrum of allergens in ragweed and mugwort pollen. Int Arch Allergy Immunol 138: 337–346. 16254437 10.1159/000089188

[9] Gadermaier G, Dedic A, Obermeyer G, Frank S, Himly M, Ferreira F (2004) Biology of weed pollen allergens. Curr Allergy Asthma Rep 4: 391–400. 15283880 10.1007/s11882-004-0090-5

[10] Leonard R, Wopfner N, Pabst M, Stadlmann J, Petersen BO, Duus JO, et al (2010) A new allergen from ragweed (Ambrosia artemisiifolia) with homology to Art v 1 from mugwort. J Biol Chem 285: 27192–27200. 10.1074/jbc.M110.127118 20576600 PMC2930718

[11] Bouley J, Groeme R, Le Mignon M, Jain K, Chabre H, Bordas-Le Floch V, et al (2015) Identification of the cysteine protease Amb a 11 as a novel major allergen from short ragweed. J Allergy Clin Immunol In press. 10.1016/j.jaci.2015.03.001 25865353

[12] Lopez-Casado G, Covey PA, Bedinger PA, Mueller LA, Thannhauser TW, Zhang S, et al (2012) Enabling proteomic studies with RNA-Seq: The proteome of tomato pollen as a test case. Proteomics 12: 761–774. 10.1002/pmic.201100164 22539427

[13] Schulten V, Greenbaum JA, Hauser M, McKinney DM, Sidney J, Kolla R, et al (2013) Previously undescribed grass pollen antigens are the major inducers of T helper 2 cytokine-producing T cells in allergic individuals. Proc Natl Acad Sci U S A 110: 3459–3464. 10.1073/pnas.1300512110 23401558 PMC3587276

[14] Evans VC, Barker G, Heesom KJ, Fan J, Bessant C, Matthews DA (2012) De novo derivation of proteomes from transcriptomes for transcript and protein identification. Nat Methods 9: 1207–1211. 10.1038/nmeth.2227 23142869 PMC3581816

[15] Campbell BC, Gilding EK, Timbrell V, Guru P, Loo D, Zennaro D, et al (2015) Total transcriptome, proteome, and allergome of Johnson grass pollen, which is important for allergic rhinitis in subtropical regions. J Allergy Clin Immunol 135: 133–142. 10.1016/j.jaci.2014.06.034 25129679

[16] Zhang P, Dreher K, Karthikeyan A, Chi A, Pujar A, Caspi R, et al (2010) Creation of a genome-wide metabolic pathway database for Populus trichocarpa using a new approach for reconstruction and curation of metabolic pathways for plants. Plant Physiol 153: 1479–1491. 10.1104/pp.110.157396 20522724 PMC2923894

[17] Chae L, Kim T, Nilo-Poyanco R, Rhee SY (2014) Genomic signatures of specialized metabolism in plants. Science 344: 510–513. 10.1126/science.1252076 24786077

[18] Kanter U, Heller W, Durner J, Winkler JB, Engel M, Behrendt H, et al (2013) Molecular and immunological characterization of ragweed (Ambrosia artemisiifolia L.) pollen after exposure of the plants to elevated ozone over a whole growing season. PLoS One 8: e61518 10.1371/journal.pone.0061518 23637846 PMC3630196

[19] El Kelish A, Zhao F, Heller W, Durner J, Winkler JB, Behrendt H, et al (2014) Ragweed (Ambrosia artemisiifolia) pollen allergenicity: SuperSAGE transcriptomic analysis upon elevated CO2 and drought stress. BMC Plant Biol 14: 176 10.1186/1471-2229-14-176 24972689 PMC4084800

[20] Nandy A, Augustin S, Wald M, Pump L, Hermann A, Treder S, et al (2013) Recombinant Major Ragweed Allergen Amb a 1: Physicochemical Characterization and Immunologic Comparison of Five Recombinant Ragweed Isoallergens Amb a 1.01 to Amb a 1.05. J Allergy Clin Immunol 131: AB16.

[21] Nandy A, Augustin S, Mitulski L, Cromwell O (2011) Isoallergen Analysis of Pectate Lyases (Amb a 1 and Amb a 2) from Commercial Short Ragweed Pollen. J Allergy Clin Immunol 127: AB168.

[22] Augustin S, Wald M, Asero R, Reese G, Klysner S, Nandy A (2013) Assessment of Amb a 1 isoallergens as basis for development of a recombinant ragweed immunotherapeutic vaccine. Allergy 68: 111.

[23] Adolphson C, Goodfriend L, Gleich GJ (1978) Reactivity of ragweed allergens with IgE antibodies. Analyses by leukocyte histamine release and the radioallergosorbent test and determination of cross-reactivity. J Allergy Clin Immunol 62: 197–210. 81221 10.1016/0091-6749(78)90208-7

[24] Asero R, Bellotto E, Ghiani A, Aina R, Villalta D, Citterio S (2014) Concomitant sensitization to ragweed and mugwort pollen: who is who in clinical allergy? Ann Allergy Asthma Immunol 113: 307–313. 10.1016/j.anai.2014.06.009 25053399

[25] Ghosh B, Perry MP, Rafnar T, Marsh DG (1993) Cloning and expression of immunologically active recombinant Amb a V allergen of short ragweed (Ambrosia artemisiifolia) pollen. J Immunol 150: 5391–5399. 7685794

[26] Hiller KM, Lubahn BC, Klapper DG (1998) Cloning and expression of ragweed allergen Amb a 6. Scand J Immunol 48: 26–36. 9714407 10.1046/j.1365-3083.1998.00355.x

[27] Hoffmann-Sommergruber K (2000) Plant allergens and pathogenesis-related proteins. What do they have in common? Int Arch Allergy Immunol 122: 155–166. 10899758 10.1159/000024392

[28] Midoro-Horiuti T, Brooks EG, Goldblum RM (2001) Pathogenesis-related proteins of plants as allergens. Ann Allergy Asthma Immunol 87: 261–271. 11686417 10.1016/S1081-1206(10)62238-7

[29] Sinha M, Singh RP, Kushwaha GS, Iqbal N, Singh A, Kaushik S, et al (2014) Current overview of allergens of plant pathogenesis related protein families. ScientificWorldJournal 2014: 543195 10.1155/2014/543195 24696647 PMC3947804

[30] Conti A, Giuffrida MG, Hoffmann-Sommergruber K, Wagner S, Amato S, Mistrello G, et al (2007) Identification of latex UDP glucose pyrophosphorylase (Hev b UDPGP) as a novel cause of latex fruit allergy syndrome. Eur Ann Allergy Clin Immunol 39: 116–118. 17523384

[31] Radauer C, Bublin M, Wagner S, Mari A, Breiteneder H (2008) Allergens are distributed into few protein families and possess a restricted number of biochemical functions. J Allergy Clin Immunol 121: 847–852. 10.1016/j.jaci.2008.01.025 18395549

[32] Fonseca C, Planchon S, Pinheiro C, Renaut J, Ricardo CP, Oliveira MM, et al (2014) Maize IgE binding proteins: each plant a different profile? Proteome Sci 12: 17 10.1186/1477-5956-12-17 24650160 PMC3999935

[33] Hur GY, Park HJ, Kim HA, Ye YM, Park HS (2008) Identification of Dioscorea batatas (sanyak) allergen as an inhalant and oral allergen. J Korean Med Sci 23: 72–76. 10.3346/jkms.2008.23.1.72 18303202 PMC2526494

[34] Zhao F, Elkelish A, Durner J, Lindermayr C, Winkler JB, Ruëff F, et al (2015) Common ragweed (Ambrosia artemisiifolia L.): Allergenicity and molecular characterisation of pollen after plant exposure to elevated NO_2_ . Plant Cell Environ 10.1111/pce.12601 26177592

[35] Chan T-F, Ji K-M, Yim AK-Y, Liu X-Y, Zhou J-W, Li R-Q, et al (2015) The draft genome, transcriptome, and microbiome of Dermatophagoides farinae reveal a broad spectrum of dust mite allergens. J Allergy Clin Immunol 135: 539–548. 10.1016/j.jaci.2014.09.031 25445830

