# Supplementary material for: Identification of Novel Short Ragweed Pollen Allergens Using Combined Transcriptomic and Immunoproteomic Approaches
Source: PLoS One. 2015 Aug 28;10(8):e0136258. doi: 10.1371/journal.pone.0136258 (PMC4552831; doi:10.1371/journal.pone.0136258)
Supplement: S1 File — (DOCX) [file pone.0136258.s001.docx]

**Supplementary materials**

**In-solution mass spectrometry (MS) analyses**

Proteins from short ragweed pollen extracts with or without protein level normalization (Proteominer kit, Bio-Rad, Marne-La-Coquette, France) were precipitated with the PerfectFocus kit (Agro-Bio, La Ferté Saint Aubin, France) and resuspended in 8 M urea, pH 8. Sample concentrations were determined using a Bradford assay (Bio-Rad). Subsequently, 10 µL of samples (20 µg) were mixed with 2 µL of a 1 % (w/v) ProteaseMax (Promega, Lyon, France) solution in 50 mM ammonium bicarbonate. Disulfide bridge reduction was performed by adding 1 µL of a 250 mM dithiothreitol solution followed by incubation for 20 min at 37°C. After cooling down at room temperature, cysteine alkylation was carried out using 1 µL of a 500 mM iodoacetamide solution after a 30 min incubation in the dark. Subsequently, 81.5 µL of 50 mM ammonium bicarbonate, 1 µL of 100 mM CaCl_2_ and 2 µL of a 0.5 µg/µL trypsin (Promega) solution were added successively. After a 3 h incubation at 37°C, enzymatic digestion was quenched by adding 1.5 µL of formic acid (FA). Peptide samples were then centrifuged at 13,000 *g* for 10 min to remove the excess of degraded surfactant.

Nano-LC-MS/MS analyses were performed using an Impact HD mass spectrometer (Bruker Daltonics, Wissembourg, France) coupled to an Ultimate 3000 RS nano LC system (Thermo Fisher Scientific, Villebon-sur-Yvette, France). Digested samples (4 µg) were first desalted in-line for 10 minutes using a C18 pre-column (Acclaim PepMap100 nanoViper 5 µm, 100 Å, 100 µm i.d x 2 cm; Thermo Scientific) using a loading buffer (0.1 % FA / 2 % ACN) with a flow rate of 15 µL/min. Chromatographic separations were performed at 50°C using a C18 column (Acclaim PepMapRSLC nanoViper, 5 µm, 100 Å, 75 µm i.d. x 25 cm; Thermo Scientific) with a flow rate of 450 nL/min. Peptides were eluted using an acetonitrile (CAN) gradient over 238 min directly in the mass spectrometer equipped with a CaptiveSpray source (Bruker Daltonics). Acquisitions were performed in positive mode with end plate offset and capillary voltages set at -500 and 1,400 V, respectively. MS spectra were acquired over the 50-2,000 m/z range with a scan rate of 2 Hz. MS/MS spectra were acquired using the Intensity Dependent Acquisition Speed (IDAS) mode with a MS-MS/MS cycle time fixed at 7 s, a trigger threshold set at 2x10^3^ counts (cts) and an acquisition speed defined as follows: 4 Hz for precursor intensity below 4x104 cts and 16 Hz for precursor intensity above 1x10^6^ cts. An active exclusion list was enabled and precursor ions were excluded after one spectrum for 120 s.

**In-gel MS analyses**

Following Sypro Ruby staining (Life Technologies), spots of interest were excised from 2D‑gels using an EXquest spot cutter (Bio-Rad) and then submitted to tryptic digestion. Briefly, spots were first destained with a 50 % ACN / 50 mM ammonium bicarbonate solution and then dehydrated using ACN. Gel pieces were then rehydrated with 20 µL of a 10 ng/µL trypsin solution prepared in 0.01 % (w/v) ProteaseMax / 50 mM Ammonium bicarbonate. After a 5 min incubation on ice, 30 µL of a 0.01 % (w/v) ProteaseMax solution in 50 mM Ammonium bicarbonate were added. After 1h at 50°C, the enzymatic digestion was quenched by adding 1 µL of FA. Digests were then centrifuged at 13,000 *g* for 10 min to remove the excess of degraded surfactant. Digested samples were analysed by nano LC‑MS/MS as described above, except for the ACN gradient which was reduced to 18 min, the MS-MS/MS cycle time fixed at 3 s and MS/MS acquisition speed set at 1 Hz for precursor intensity below 4x10^4^ cts and 8 Hz for precursor intensity above 1x10^6^ cts.

**Protein identification**

Protein/peptide identifications were performed using the PEAKS software (Bioinformatics Solutions Inc., PEAKS Studio version 7). All searches were done against the Transcriptome-Derived Proteome (TDP) database supplemented with Amb a 4, Amb a 5, Amb a 6 and Amb a 8.0102 sequences recorded in the IUIS database. The database search parameters included up to 1 missed cleavage, 1 non-specific cleavage, a precursor ion mass tolerance of 10 ppm, a false discovery rate ≤ 1 % at peptide level and a fragment ion mass tolerance of 0.05 Da. Carbamidomethylation of cysteine (+57.0214) was set as a fixed modification. No variable modification was allowed. Only proteins identified with a minimum of 2 peptides sequenced including at least 1 unique peptide, were taken into account. All proteins identified with the same set of peptides were grouped by the PEAKS algorithm and only the highest scoring hit of each protein group was conserved in the protein list. In case of multiple-frame translations due to frameshift(s) in nucleotide sequences, the respective numbers of peptides were added.
