# Supplementary material for: Identification of Novel Short Ragweed Pollen Allergens Using Combined Transcriptomic and Immunoproteomic Approaches
Source: PLoS One. 2015 Aug 28;10(8):e0136258. doi: 10.1371/journal.pone.0136258 (PMC4552831; doi:10.1371/journal.pone.0136258)
Supplement: S1 Table — A short ragweed pollen extract was analyzed by LC-MS/MS (prior and after protein abundance normalization). Protein identification was performed using the PEAKS software and the annotated TDP database. Only the master proteins in each protein group are reported. The transcript number, total number of peptides and number of unique peptides sequenced are provided as well as, when available, the Uniprot entry and annotation of a known homolog. (PDF) [file pone.0136258.s002.pdf]

Allergen name	Transcript number	Sequenced peptides	Unique sequenced peptides	Homolog uniprot entry	Annotation
	isotig04531	57	55	G7L857_MEDTR	Galactose oxidase
	isotig04683	47	45	B9HEB7_POPTR	Predicted protein
	isotig00904	45	1	G3FLW0_MANIN	Actin 4
	isotig04820	43	14	TBB1_MAIZE	Tubulin beta-1 chain
	isotig00400	41	1	G5EN35_9ASTR	Actin
	isotig04668	41	2	G5EN35_9ASTR	Actin
	isotig05518	41	38	IPYR_SOLTU	Soluble inorganic pyrophosphatase
	isotig04431	38	37	B9RCJ8_RICCO	ATP binding protein putative
	isotig04734	38	9	TBB1_MAIZE	Tubulin beta-1 chain
Amb a 1.02	isotig01677	37	26	E1XUL3_AMBAR	Putative pectate lyase
	isotig01609	36	36	Q2WFK8_9ASTR	Fructose-bisphosphate aldolase
	isotig03176	36	34	B9RET0_RICCO	Protein transport protein Sec24C putative
Amb a 1.03	isotig01670	35	25	E1XUL4_AMBAR	Putative pectate lyase
	isotig03205	35	12	B9GL62_POPTR	Acyl-coA synthetase family protein
	isotig04714	35	16	D7KYQ2_ARALL	Alpha-1 tubulin
	isotig04511	34	33	B9IAP6_POPTR	Fimbrin-like family protein
Amb a 1.01	isotig01671	33	29	MPA11_AMBAR	Pollen allergen Amb a 1.1
	isotig04525	33	32	G7L857_MEDTR	Galactose oxidase
	isotig04571	33	31	A5CKE3_SOLLC	Rab-GDP dissociation inhibitor
	isotig03294	32	32	F6M8J8_POPDE	UDP-glucose pyrophosphorylase
	isotig03296	32	28	A5ATJ7_VITVI	Pectinesterase
	isotig03178	32	32	B9H9T9_POPTR	Villin 2 family protein
	isotig04479	30	30	B9GGI8_POPTR	Predicted protein
	isotig04439	29	29	D7U9X3_VITVI	Pectinesterase
	isotig04480	29	28	A6YM33_RICCO	Phosphoenolpyruvate carboxylase
	isotig04617	29	11	B9R6R7_RICCO	Tubulin alpha chain putative
	isotig03416	28	28	B9ST19_RICCO	Serine/threonine protein phosphatase 2a regulatory subunit A putative
Amb a 1.04	isotig01678	27	23	MPA14_AMBAR	Pollen allergen Amb a 1.4
	isotig02173	27	23	Q287V1_9BRAS	Putative plasma membrane ATPase
Amb a 1.05	isotig04729	26	25	MPAA2_AMBAR	Pollen allergen Amb a 2
	isotig03179	26	25	Q5K4L5_TOBAC	Villin 1
Amb a 1-like	isotig01676	25	24	MPA11_AMBAR	Pollen allergen Amb a 1.1
	isotig03369	25	3	B9GIF5_POPTR	Predicted protein
	isotig04535	25	25	O82722_NICSY	ATP synthase subunit beta
	isotig04752	25	24	G7L857_MEDTR	Galactose oxidase
	isotig05812	25	23	PLDA1_CYNCA	Phospholipase D alpha 1
Amb a 1-like	isotig04702	24	22	A0PJ16_ARTVU	Amb a 1-like protein
	isotig05285	24	24	PLDA1_CYNCA	Phospholipase D alpha 1
	isotig04627	23	6	A9P7T6_POPTR	Tubulin alpha-4 chain
	isotig02118	22	17	Q2KN24_AMBAR	Profilin
	isotig04499	22	20	Q6UN74_TOBAC	Ntp101
	isotig04527	22	22	Q5BN15_PETHY	Pyruvate decarboxylase 2
	isotig04622	22	22	Q9MBB7_SALGI	Polygalacturonase
	isotig04528	21	21	B9RKI6_RICCO	Malic enzyme
	isotig05036	21	21	B9SP64_RICCO	Phosphoglucomutase putative
	isotig03212	20	20	B9RZB3_RICCO	Phosphatidylinositol 4-kinase putative
	isotig03244	20	8	Q6Q8A6_TOBAC	Hexokinase 1a
	isotig04442	20	18	B9GN25_POPTR	Pleckstrin homology domain-containing family protein
	isotig04419	20	18	B9S0J4_RICCO	Pleiotropic drug resistance protein putative

Allergen name	Transcript number	Sequenced peptides	Unique sequenced peptides	Homolog uniprot entry	Annotation
	isotig04498	20	19	B9GZR0_POPTR	Pectinesterase
	isotig04633	20	20	Q2WFI2_9ASTR	Isocitrate dehydrogenase [NADP]
	isotig04735	20	20	A1KXD9_LACSA	26S proteasome subunit 7-like protein
	isotig04924	20	20	Q5I6D6_9ROSI	Sinapyl alcohol dehydrogenase-like protein
	isotig04586	19	19	B9SDN2_RICCO	Calcium-dependent protein kinase putative
	isotig07102	19	19	B9HSX0_POPTR	Predicted protein
	isotig05375	19	5	G7K096_MEDTR	ADP-ribosylation factor
	isotig06350	19	19	B9SRJ0_RICCO	Chemocyanin putative
Amb a 1-like	isotig03262	18	13	A0PJ16_ARTVU	Amb a 1-like protein
Amb a 1-like	isotig04731	18	13	A0PJ16_ARTVU	Amb a 1-like protein
	isotig02537	18	17		Villin 2 family protein
	isotig03089	18	6	G5EN35_9ASTR	Actin
	isotig03245	18	6	Q6Q8A6_TOBAC	Hexokinase 1a
	isotig04681	18	18	B9SRZ2_RICCO	Isocitrate dehydrogenase putative
	isotig04898	18	18	Q2PZA5_CUCME	Putative alcohol dehydrogenases
	isotig04921	18	18	D7MN90_ARALL	Aldose 1-epimerase family protein
	isotig05412	18	16	Q6J205_TROMA	14-3-3 protein
Amb a 8.01		17	12	Q2KN23_AMBAR	Profilin
	isotig03862	17	3	Q677H6_HYAOR	ADP-ribosylation factor
	isotig04521	17	15	Q6UN74_TOBAC	Ntp101
	isotig04567	17	16	B9H9N5_POPTR	Calcium dependent protein kinase 2
	isotig04801	17	17	CYSEP_PHAVU	Vignain
	isotig05083	17	14	G7JHI0_MEDTR	14-3-3-like protein
	isotig05175	17	17	G7L3T9_MEDTR	Cell division cycle protein-like protein
	isotig02197	16	16	B9RMQ3_RICCO	Alpha-L-fucosidase 2 putative
	isotig05399	16	12	A9PCE5_POPTR	14-3-3-like protein GF14 iota
	isotig04846	16	16	Q6RUF8_SOYBN	Glycerol kinase
	isotig04707	15	15	Q9MBB7_SALGI	Polygalacturonase
	isotig04751	15	15	B9RKL6_RICCO	Galactose oxidase putative
	isotig04858	15	15	B9HBY5_POPTR	Predicted protein
	isotig06807	15	2	B9IPS6_POPTR	Predicted protein
	isotig03364	14	13	LOR15_ARATH	Protein LURP-one-related 15
	isotig03486	14	14	A7DX12_LOTJA	A-type carbonic anhydrase
	isotig04441	14	13	B9SN36_RICCO	Protein SEY1 putative
	isotig04492	14	14	G7K028_MEDTR	Phosphatidylinositide phosphatase SAC1
	isotig04784	14	14	I1W1T8_PRUPE	Cytosolic invertase 1
	isotig04791	14	14	Q1M0P0_POPTO	UDP-glucuronic acid decarboxylase 3
	isotig04843	14	14	I6LNT9_HELAN	Phosphoglycerate kinase
	isotig03226	13	9	G7JCD0_MEDTR	Plasma membrane H+ ATPase
	isotig03256	13	3	I7FJJ2_TOBAC	MAP kinase
	isotig03399	13	13	D7MLU4_ARALL	Phosphofructokinase family protein
	isotig04416	13	13	Q8S912_TOBAC	Microtubule bundling polypeptide TMBP200
	isotig04629	13	13	B9RRN0_RICCO	Protein transport protein sec23 putative
	isotig04793	13	13	Q5M9V4_TOBAC	ATP synthase subunit alpha
	isotig04987	13	11	B9GY91_POPTR	Serine/threonine-protein phosphatase
	isotig01777	12	1	PLY59_SOLLC	Probable pectate lyase P59
	isotig01779	12	1	PLY59_SOLLC	Probable pectate lyase P59
	isotig03214	12	7	B9GN25_POPTR	Pleckstrin homology domain-containing family protein
	isotig03246	12	12	B9SIF7_RICCO	Diacylglycerol kinase alpha putative

Allergen name	Transcript number	Sequenced peptides	Unique sequenced peptides	Homolog uniprot entry	Annotation
	isotig03312	12	10	Q9S7F7_FRAAN	Cytosolic ascorbate peroxidase
	isotig04628	12	12	Q6VAA8_STERE	UDP-glycosyltransferase 91D1
	isotig04470	12	12	B9H7Y5_POPTR	Predicted protein
	isotig04781	12	12	B9T1B9_RICCO	Patellin-4 putative
	isotig04800	12	12	B9SAG9_RICCO	Receptor serine-threonine protein kinase putative
	isotig05000	12	10	I1KXM8_SOYBN	Serine/threonine-protein phosphatase
	isotig05077	12	12	D7TIZ5_VITVI	Pyruvate kinase
	isotig05344	12	9	B9R9J4_RICCO	Fructokinase putative
	isotig05536	12	4	Q40203_LOTJA	RAB1C
	isotig05693	12	11	CAPPB_FLATR	Phosphoenolpyruvate carboxylase 1
	isotig07010	12	6	F2CVV2_HORVD	Predicted protein
Amb a 3-like	isotig04817	11	11	MPAA3_AMBEL	Pollen allergen Amb a 3
Amb a 6.01		11	11	NLTP6_AMBAR	Non-specific lipid-transfer protein
	isotig01864	11	11	Q2KM81_ARTVU	Polcalcin
	isotig05139	11	11	B9GF67_POPTR	Predicted protein
	isotig02868	11	11	E6NU09_9ROSI	JHL07K02.9 protein
	isotig04448	11	11	A9ZM29_SOYBN	Oxysterol-binding protein
	isotig05401	11	11	B9RAG9_RICCO	AMP-activated protein kinase gamma regulatory subunit putative
	isotig06452	11	11	H6WJJ6_9ROSI	Pathogenesis-related protein 17
Amb a 8.01	isotig06625	10	8	PROF3_AMBAR	Profilin-3
Amb a 11	isotig04855	10	10	V5LU01_AMBAR	Amb a 11 Cysteine protease
	isotig02260	10	10	Q41724_ZINEL	TED2
	isotig02326	10	10	B9SSY3_RICCO	Type II inositol 5-phosphatase putative
	isotig03274	10	10	Q5DUH0_CAPCH	Glutathione S-transferase GST1
	isotig03299	10	10	B9HAS7_POPTR	Pectinesterase
	isotig03448	10	10	B9GKZ1_POPTR	Transducin family protein
	isotig04546	10	10	Q3HLN2_TOBAC	Sucrose-phosphate synthase isoform B
	isotig04547	10	10	B9SR46_RICCO	2-oxoglutarate dehydrogenase putative
	isotig04570	10	10	B9RM62_RICCO	Aspartic proteinase Asp1 putative
	isotig05369	10	9	B9H4T8_POPTR	Ras-related GTP-binding family protein
	isotig04703	10	10	PLY59_SOLLC	Probable pectate lyase P59
	isotig05151	10	6	B9HBS5_POPTR	Predicted protein
	isotig04892	10	10	B9S8G9_RICCO	Zinc finger protein putative
	isotig05063	10	10	D7LD44_ARALL	Esterase/lipase/thioesterase family protein
	isotig05203	10	9	LOR15_ARATH	Protein LURP-one-related 15
	isotig07197	10	7	B9I610_POPTR	Predicted protein
	isotig05874	10	10	XYL2_MEDSV	Beta-xylosidase/alpha-L-arabinofuranosidase 2
	isotig06357	10	10	O24548_VIGUN	Type IIIa membrane protein cp-wap13
	isotig06051	10	10	F2EBF9_HORVD	Predicted protein
	isotig02653	9	9	No hit	
	isotig03297	9	6	I1MK76_SOYBN	Pectinesterase
	isotig03458	9	5	Q6T8C9_HELAN	Putative alpha-soluble NSF attachment protein
	isotig03712	9	9	Q5KQH5_ORYSJ	putative 2 3-bisphosphoglycerate-independent phosphoglycerate mutase'
	isotig03920	9	6	G1FCF5_9ROSI	Fructokinase
	isotig04427	9	7	B9S0J4_RICCO	Pleiotropic drug resistance protein putative
	isotig05050	9	3	B9GXB4_POPTR	Predicted protein
	isotig04776	9	9	Q96383_CENCA	Aspartic proteinase
	isotig04927	9	9	A0AQQ6_HORVU	Inositol phosphate kinase
	isotig05108	9	9	B9RPP7_RICCO	DUF26 domain-containing protein 2 putative

Allergen name	Transcript number	Sequenced peptides	Unique sequenced peptides	Homolog uniprot entry	Annotation
	isotig03576	9	9	B9HU21_POPTR	Predicted protein
	isotig05306	9	9	B9RZW7_RICCO	Dihydrolipoyl dehydrogenase
	isotig05511	9	8	J7F320_CAMSI	Eukaryotic translation initiation factor 5A4
	isotig05846	9	9	D7TMQ2_VITVI	Citrate synthase
	isotig04447	9	9	B9IHR4_POPTR	Phosphatidylinositol-4-phosphate 5-kinase family protein
	isotig05984	9	9	A6GVD5_ARTVU	Art v 2 allergen
	isotig02675	9	1	B9MT67_POPTR	Predicted protein
	isotig04874	9	9	B9NE02_POPTR	Predicted protein
	isotig07303	9	1	Q9SXT5_CICAR	Rab-type small GTP-binding protein
	isotig06976	9	9	F2DAY3_HORVD	Lactoylglutathione lyase
	isotig03003	8	8	G7I824_MEDTR	Glucose and ribitol dehydrogenase
	isotig05115	8	4	B9GG35_POPTR	Predicted protein
	isotig03119	8	8	B9RC84_RICCO	Pollen-specific protein SF3 putative
	isotig03215	8	3	B9GN25_POPTR	Pleckstrin homology domain-containing family protein
	isotig03970	8	1	I0CC93_9CARY	Type II peroxiredoxin
	isotig03971	8	1	A3FPF4_NELNU	Thioredoxin-dependent peroxidase
	isotig04425	8	8	Q5MNW6_GOSHI	Kinesin
	isotig04474	8	8	G7JS80_MEDTR	Leucine-rich repeat receptor-like protein kinase
	isotig04548	8	8	A4GU96_GOSHI	Kinesin-related protein
	isotig07641	8	8	B9H3U7_POPTR	Predicted protein
	isotig05149	8	7	B9H9Z0_POPTR	DEAD box RNA helicase family protein
	isotig05141	8	8	D2D330_GOSHI	Lactoylglutathione lyase
	isotig05163	8	6	Q5DK69_SOLLC	Pollen-specific kinase partner protein
	isotig05365	8	8	B9SI95_RICCO	Early nodulin 55-2 putative
	isotig05517	8	8	Q9M6K5_TARER	Isopentenyl pyrophosphate:dimethyllallyl pyrophosphate isomerase
	isotig05907	8	1	Q45W77_ARAHY	Ubiquitin-conjugating enzyme 1
	isotig02381	8	6	B9IJL9_POPTR	Predicted protein
	isotig04937	8	8	B9ILA9_POPTR	Phosphatase 2C family protein
	isotig05985	8	8	B9IPD3_POPTR	S-adenosyl-L-methionine:carboxyl methyltransferase family protein
	isotig06099	8	8	A6YM33_RICCO	Phosphoenolpyruvate carboxylase
	isotig06493	8	8	B9MTX7_POPTR	Haloacid dehalogenase-like hydrolase family protein
	isotig06173	8	8	Q6ZFF2_ORYSJ	Os08g0482600 protein
	isotig06364	8	1	Q45W77_ARAHY	Ubiquitin-conjugating enzyme 1
	isotig02870	8	7	B9MXN5_POPTR	Predicted protein
	isotig06444	8	8	PSB1_PETHY	Proteasome subunit beta type-1
	isotig06769	8	8	D6BRD7_9ROSI	Universal stress protein
	isotig01832	8	8	B9N470_POPTR	Predicted protein
	isotig07006	8	8	B3F8F4_SOLTU	Glutaredoxin
	contig24397	7	7	No hit	
	isotig00331	7	7	VA726_ARATH	Putative vesicle-associated membrane protein 726
	isotig01811	7	2	CALM_HELAN	Calmodulin
	isotig02442	7	7	B6TIJ3_MAIZE	Thioredoxin
	isotig05271	7	7	B9GKD5_POPTR	Predicted protein
	isotig03338	7	7	Q7GDU7_ORYSJ	Cysteine endopeptidase
	isotig03426	7	7	G7JFL5_MEDTR	ATP phosphoribosyltransferase
	isotig03497	7	7	D7UAT7_VITVI	Uridine kinase
	isotig03870	7	6	ENO1_HEVBR	Enolase 1
	isotig02824	7	1	B9GRN8_POPTR	Predicted protein
	isotig04459	7	7	D7MB86_ARALL	Kinase family protein

Allergen name	Transcript number	Sequenced peptides	Unique sequenced peptides	Homolog uniprot entry	Annotation
	isotig04514	7	2	B9HPN3_POPTR	Calcium dependent protein kinase 5
	isotig04526	7	7	A1KXE1_LACSA	Pectin acetylesterase
	isotig04657	7	7	B9RTM1_RICCO	Protein kinase atmrk1 putative
	isotig04662	7	7	Q9SSZ1_HELAN	Aspartic proteinase
	isotig04798	7	3	B9HPN3_POPTR	Calcium dependent protein kinase 5
	isotig04952	7	7	B9RB80_RICCO	Peroxidase 65 putative
	isotig05118	7	7	Q40495_TOBAC	N-ethylmaleimide sensitive fusion protein
	isotig05126	7	7	B9T5A1_RICCO	Cholinephosphate cytidylyltransferase putative
	isotig05144	7	7	O22406_PETCR	Glucose-6-phosphate 1-dehydrogenase
	isotig03055	7	7	B9HTD1_POPTR	Transferase family protein
	isotig05454	7	7	B3TLV8_ELAGV	Ubiquitin-conjugating enzyme family protein
	isotig04588	7	7	B9I9L6_POPTR	CTP synthase
	isotig05821	7	7	B9RES5_RICCO	Calcium ion binding protein putative
	isotig05027	7	7	B9IGR3_POPTR	Predicted protein
	isotig06174	7	3	ADF7_ARATH	Actin-depolymerizing factor 7
	isotig06378	7	7	ERG3_ORYSJ	Elicitor-responsive protein 3
	isotig06387	7	7	D6BRD7_9ROSI	Universal stress protein
	isotig04694	7	7	D7MT53_ARALL	Predicted protein
	isotig07466	7	7	F2E7L1_HORVD	Histone H4
	isotig01197	6	4	B9H0Q2_POPTR	Adenylyl cyclase-associated protein
	isotig02205	6	6	D6BR66_9ROSI	Glutathione S-transferase omega
	isotig03551	6	6	A9PDU2_POPTR	Predicted protein
	isotig02609	6	4	Q8H2B6_TOBAC	Pollen specific actin-depolymerizing factor 2
	isotig02635	6	6	B9RMQ3_RICCO	Alpha-L-fucosidase 2 putative
	isotig02722	6	6	B9HCD3_POPTR	Autoinhibited H+ ATPase
	isotig05071	6	6	B9GEN8_POPTR	Ribose 5-phosphate isomerase family protein
	isotig03180	6	5	B9R839_RICCO	ATP binding protein putative
	isotig03196	6	4	E5GB47_CUCME	Eukaryotic peptide chain release factor subunit
	isotig04639	6	6	B9GJK1_POPTR	Dehydration-responsive family protein
	isotig03218	6	6	J7FH09_MESCR	Sucrose non-fermenting 1
	isotig03459	6	2	Q6T8C9_HELAN	Putative alpha-soluble NSF attachment protein
	isotig03517	6	4	Q9XGI6_SOLLC	Expansin9
	isotig03538	6	2	C9E9M0_PHAVU	RabA2
	isotig03539	6	4	RAA1F_ARATH	Ras-related protein RABA1f
	isotig03608	6	2	PP12_TOBAC	Serine/threonine-protein phosphatase PP1 isozyme 2
	isotig03653	6	6	B9SI95_RICCO	Early nodulin 55-2 putative
	isotig03660	6	6	D7MPW2_ARALL	Vesicle-associated membrane family protein
	isotig05704	6	3	B9GXB4_POPTR	Predicted protein
	isotig04504	6	6	Q6KC53_NICPL	Calcium-dependent protein kinase 17
	isotig04506	6	6	Q2PYW3_SOLTU	Vacuolar sorting receptor protein PV72-like protein
	isotig04517	6	6	B9SFZ6_RICCO	Phosphatidylinositol-3 4 5-trisphosphate 3-phosphatase and dual-specificity protein phosphatase PTEN putative
	isotig04520	6	6	B9RJX8_RICCO	Restin putative
	isotig04551	6	3	B0FC97_9FABA	Calcium-dependent protein kinase
	isotig04581	6	4	D7LI11_ARALL	Phospholipase D beta 1
	isotig04711	6	6	B5M9E5_SOLLC	Beta-glucosidase 08
	isotig04746	6	2	PP12_TOBAC	Serine/threonine-protein phosphatase PP1 isozyme 2
	isotig04840	6	6	Q70Z24_TOBAC	Protein kinase CK2 alpha chain
	isotig04907	6	6	B9RWX1_RICCO	ATP binding protein putative

Allergen name	Transcript number	Sequenced peptides	Unique sequenced peptides	Homolog uniprot entry	Annotation
	isotig04981	6	6	D7MNZ2_ARALL	Glycerophosphoryl diester phosphodiesterase family protein
	isotig04869	6	6	B9I192_POPTR	Myosin heavy chain-related family protein
	isotig06567	6	6	B9I610_POPTR	Predicted protein
	isotig05534	6	6	I1QY51_ORYGL	Coatomer subunit beta
	isotig03542	6	4	B9IA37_POPTR	Predicted protein
	isotig05686	6	6	D7LHK4_ARALL	Kinase
	isotig05767	6	6	CALX_HELTU	Calnexin homolog
	isotig05831	6	6	H6VNP1_LYCBA	LAT52-like protein
	isotig05906	6	6	D3JX88_PANGI	Glutamate decarboxylase
	isotig05941	6	6	B3GPG7_ORYSJ	Spermidine synthase 3
	isotig06082	6	6	Q84ZX6_SOLLC	Sucrose-phosphatase
	isotig04721	6	6	B9MV41_POPTR	Predicted protein
	isotig06249	6	6	A3F7Q4_GOSHI	Phenylcoumaran benzylic ether reductase-like protein
	isotig04744	6	6	B9MWU0_POPTR	Acid phosphatase survival protein SurE
	isotig04912	6	6	B9N328_POPTR	Predicted protein
	isotig06571	6	6	E6NU33_9ROSI	JHL05D22.14 protein
	isotig06764	6	6	A9PBC3_POPTR	Glutaredoxin C4
	isotig06802	6	5	G7LI12_MEDTR	Actin depolymerizing factor
	isotig05400	6	6	D7M708_ARALL	Predicted protein
Amb a 5.01		5	5	MPAA5_AMBEL	Pollen allergen Amb a 5
	contig22391	5	5	G7LBH9_MEDTR	Protein vip1
	isotig01202	5	3	B9H0Q2_POPTR	Adenylyl cyclase-associated protein
	isotig01412	5	5	Q8RXH5_TOBAC	Osmotic stress-activated protein kinase
	isotig01868	5	3	I6XNH4_LINUS	Putative actin-depolymerizing factor 12
	isotig05866	5	5	A9PCA9_POPTR	NPL4 family protein
	isotig02120	5	5	H2L0T2_HELAN	Preproalbumin PawS1
	isotig02184	5	5	I3SK85_LOTJA	Glutathione peroxidase
	isotig02228	5	1	B9S5V9_RICCO	Casein kinase putative
	isotig02259	5	5	A1L4Y4_ARATH	At5g61510
	isotig02499	5	5	A5BTZ8_VITVI	Annexin
	isotig02543	5	5	B9RYE9_RICCO	Polyadenylate-binding protein putative
	isotig02551	5	5	P93666_HELAN	Leucine-rich-repeat protein
	isotig06168	5	5	A9PFY3_POPTR	Predicted protein
	isotig02674	5	4	F4IQ28_ARATH	RabGAP/TBC domain-containing protein
	isotig06303	5	5	A9PG62_POPTR	ADP-ribosylation factor family protein
	isotig03184	5	5	Q001P5_ARTAN	NADPH--cytochrome P450 reductase
	isotig03190	5	5	B9RLG2_RICCO	O-linked n-acetylglucosamine transferase ogt putative
	isotig03213	5	5	D7KE30_ARALL	Phosphatidylinositol 4-kinase
	isotig03234	5	4	B9RQ50_RICCO	Fimbrin putative
	isotig03454	5	5	G7ICJ3_MEDTR	Serine/threonine-protein phosphatase
	isotig03516	5	3	Q8LKK0_GOSHI	Alpha-expansin
	isotig03559	5	3	Q9SXT7_CICAR	Rac-type small GTP-binding protein
	isotig03642	5	5	Q719L3_PARAR	Rubber synthesis protein
	isotig03861	5	5	A5HIG2_9FABA	Succinyl-CoA ligase beta subunit
	isotig03871	5	4	D7LUC8_ARALL	Enolase
	isotig06044	5	5	B9GV21_POPTR	FH INTERACTING protein 1
	isotig04268	5	2	G8XWY8_9ASTR	Glyceraldehyde 3-phosphate dehydrogenase
	isotig06018	5	5	B9H523_POPTR	Predicted protein
	isotig04690	5	5	B9RZ67_RICCO	Expressed protein putative

Allergen name	Transcript number	Sequenced peptides	Unique sequenced peptides	Homolog uniprot entry	Annotation
	isotig04691	5	4	PLY_TOBAC	Pectate lyase
	isotig04692	5	5	METK2_TOBAC	S-adenosylmethionine synthase 2
	isotig04864	5	3	D7LI11_ARALL	Phospholipase D beta 1
	isotig04900	5	5	ATPG3_IPOBA	ATP synthase subunit gamma mitochondrial
	isotig04901	5	5	Q5D8D3_SOLLC	Acyl-coenzyme A oxidase
	isotig05154	5	5	D7MPL3_ARALL	Glycerophosphoryl diester phosphodiesterase family protein
	isotig05199	5	5	B1PVT0_TOBAC	LIM domain protein 2b
	isotig05210	5	5	G7JAD8_MEDTR	Ribonuclease P protein subunit p25
	isotig05265	5	5	G7J933_MEDTR	Endoglucanase
	isotig05278	5	2	E1XUL5_AMBAR	Putative pectate lyase
	isotig05422	5	5	D7M301_ARALL	Catalytic/ coenzyme binding protein
	isotig05483	5	2	B9S6D8_RICCO	Inorganic pyrophosphatase putative
	isotig04047	5	5	B9IA95_POPTR	Eukaryotic translation initiation factor 3 subunit H
	isotig05692	5	5	B9SP79_RICCO	ARF GTPase activator putative
	isotig05021	5	5	B9IEY8_POPTR	Leucine-rich repeat family protein
	isotig05824	5	5	A6GVD5_ARTVU	Art v 2 allergen
	isotig06073	5	5	B9SZM7_RICCO	Calcium ion binding protein putative
	isotig06125	5	5	Q9LSL3_ARATH	Oligopeptidase A
	isotig05280	5	5	B9MUG4_POPTR	Invertase/pectin methylesterase inhibitor family protein
	isotig06172	5	5	D9IL91_BRARP	Phytocystatin 5-3
	isotig06284	5	3	G8XR12_GOSHI	UDP-glucuronic acid decarboxylase 2
	isotig06287	5	5	Q6T8C6_HELAN	Initiation factor eIF4A-15
	isotig06304	5	5	Q9FS79_WHEAT	Triosephosphate isomerase
	isotig06380	5	1	B6SI29_MAIZE	Histone H2A
	isotig06573	5	5	G8HAB2_PAPSO	PLP-dependent aminotransferase
	isotig06593	5	4	LEA14_GOSHI	Late embryogenesis abundant protein Lea14-A
	isotig06734	5	5	RS13_SOYBN	40S ribosomal protein S13
	isotig06750	5	1	B6SI29_MAIZE	Histone H2A
	isotig03260	5	1	B9NAS8_POPTR	Predicted protein
	isotig03261	5	1	B9NAS8_POPTR	Predicted protein
	isotig07112	5	3	G7KP54_MEDTR	GPI-anchored protein
	isotig05463	5	5	D7M708_ARALL	Predicted protein
	isotig07264	5	5	I1SSI8_HEVBR	Thioredoxin
	isotig07447	5	1	PROF_HELAN	Profilin
	isotig07487	5	2	Q6RUQ2_DAUCA	Glyceraldehyde 3-phosphate dehydrogenase
	isotig00591	4	4	G7JXY1_MEDTR	SNAP25 homologous protein SNAP33
	isotig05628	4	2	A9PD45_POPTR	UDP-XYLOSE SYNTHASE 4 family protein
	isotig02623	4	4	B9T1M8_RICCO	Kinesin heavy chain putative
	isotig03086	4	4	B9RM70_RICCO	Translation initiation factor putative
	isotig03131	4	3	B6VAD7_BUPCH	LEA-2 protein
	isotig03204	4	4	B5LAV6_CAPAN	Putative long-chain acyl-CoA synthetase
	isotig03313	4	2	Q40589_TOBAC	Cytosolic ascorbate peroxidase
	isotig03339	4	4	CYSEP_PHAVU	Vignain
	isotig04612	4	4	B9GM16_POPTR	Uridine 5'-monophosphate synthase family protein
	isotig03782	4	2	B9RGR3_RICCO	Serine/threonine protein kinase putative
	isotig04420	4	4	B9RDF9_RICCO	Nucleotide binding protein putative
	isotig05710	4	4	B9GX99_POPTR	Cysteine proteinase inhibitor
	isotig04467	4	4	B9S097_RICCO	Polyadenylate-binding protein putative
	isotig04486	4	4	B9RM96_RICCO	Protein binding protein putative

Allergen name	Transcript number	Sequenced peptides	Unique sequenced peptides	Homolog uniprot entry	Annotation
	isotig03202	4	2	B9GXH9_POPTR	Acyl-coA synthetase family protein
	isotig04530	4	4	B9I9Z0_POPTR	Serine/threonine-protein phosphatase
	isotig04594	4	4	B9R7M4_RICCO	F-box and wd40 domain protein putative
	isotig04688	4	4	B9R8X2_RICCO	Chaperone protein dnaJ putative
	isotig04706	4	4	B9H2J6_POPTR	Cytochrome P450
	isotig04724	4	4	A7DX12_LOTJA	A-type carbonic anhydrase
	isotig04725	4	4	B9S773_RICCO	Lupus la ribonucleoprotein putative
	isotig04778	4	4	B9T3M9_RICCO	Glucan endo-1 3-beta-glucosidase putative
	isotig04783	4	4	Q6F4I8_ZINEL	Gamma-glutamylcysteine synthetase
	isotig04837	4	4	B9SWG9_RICCO	Beta-fructofuranosidase insoluble isoenzyme 1 putative
	isotig04847	4	4	F6I390_VITVI	Pectinesterase
	isotig04983	4	4	B9S7L4_RICCO	Calcium ion binding protein putative
	isotig05052	4	4	No hit	
	isotig05300	4	4	B9HSK1_POPTR	Predicted protein
	isotig05150	4	4	G7JW19_MEDTR	Vesicle-associated membrane protein
	isotig05260	4	4	B9RI05_RICCO	Proteasome subunit alpha type
	isotig05272	4	4	QPCT_ARATH	Glutaminyl-peptide cyclotransferase
	isotig04827	4	4	B9I3T0_POPTR	Predicted protein
	isotig02858	4	4	B9I5L5_POPTR	Predicted protein
	isotig05373	4	4	B9RCL0_RICCO	Na(+)/H(+) antiporter putative
	isotig05379	4	4	G7JXI6_MEDTR	26S proteasome non-ATPase regulatory subunit
	isotig07135	4	1	B9I610_POPTR	Predicted protein
	isotig05403	4	2	RB2BV_BETVU	Ras-related protein Rab2BV
	isotig05501	4	4	B9T7A1_RICCO	Ef-hand calcium binding protein putative
	isotig05662	4	4	B9R8B6_RICCO	Calcium lipid binding protein putative
	isotig05684	4	4	D1MWZ0_CITLA	GRAM domain-containing protein
	isotig05903	4	4	CYC_HELAN	Cytochrome c
	isotig02558	4	3	B9IK20_POPTR	Predicted protein
	isotig05940	4	4	MMT1_WOLBI	Methionine S-methyltransferase
	isotig06019	4	3	B9RYE9_RICCO	Polyadenylate-binding protein putative
	isotig06088	4	4	Q9FYX1_SOLLC	BAC19.5
	isotig06228	4	4	B9RFW4_RICCO	Pyruvate dehydrogenase putative
	isotig06399	4	4	Q0G879_CAMSI	Cyclophilin
	isotig06465	4	4	Q8H6C0_SILLA	Oligomycin sensitivity conferring protein
	isotig06525	4	4	F8SKC4_HEVBR	TCTP.1
	isotig07053	4	4	B9S5F1_RICCO	Proline oxidase putative
	isotig07362	4	4	PLRX1_ARATH	Pollen-specific leucine-rich repeat extensin-like protein 1
	isotig07605	4	4	I1SSI8_HEVBR	Thioredoxin
	isotig04255	3	3	A9P8N0_POPTR	Predicted protein
	isotig01124	3	3	Q1LYX3_ARATH	At5g42560
	isotig01357	3	3	Q9ZNX3_PETHY	PGPS/D7
	isotig01452	3	3	G7L1H1_MEDTR	Shaggy-related protein kinase
	isotig03085	3	3	A9P9K5_POPTR	eIF4-gamma/eIF5/eIF2-epsilon domain-containing family protein
	isotig01977	3	3	Q38678_AMBAR	Cystatin proteinase inhibitor
	isotig02092	3	3	B9RT59_RICCO	Kif4 putative
	isotig02826	3	3	D7MQT4_ARALL	Lipin family protein
	isotig04762	3	3	B9GFY8_POPTR	SEC14 cytosolic factor family protein
	isotig03043	3	3	Q3HRW8_SOLTU	60S ribosomal protein L18a
	isotig03177	3	1	B9RET0_RICCO	Protein transport protein Sec24C putative

Allergen name	Transcript number	Sequenced peptides	Unique sequenced peptides	Homolog uniprot entry	Annotation
	isotig04608	3	3	B9GJ76_POPTR	Myosin heavy chain-related family protein
	isotig03404	3	3	Q84RS1_MEDSA	ZIK1 protein
	isotig03456	3	3	B9SB24_RICCO	GTP-dependent nucleic acid-binding protein engD putative
	isotig03572	3	3	H6VNP1_LYCBA	LAT52-like protein
	isotig04682	3	3	B9GNW6_POPTR	Pleckstrin homology domain-containing family protein
	isotig03668	3	3	I1MLJ3_SOYBN	Uridine kinase
	isotig03742	3	3	B9T0Q0_RICCO	Calcium binding protein/cast putative
	isotig03830	3	3	G7LCC0_MEDTR	26S proteasome non-ATPase regulatory subunit
	isotig03831	3	3	O48844_ARATH	26S proteasome regulatory subunit
	isotig03906	3	3	F2Y9D8_LITCN	Aconitase protein
	isotig04728	3	3	B9GVL4_POPTR	Predicted protein
	isotig04278	3	2	Q94BT9_ARATH	Copper transport protein ATX1
	isotig04452	3	3	Q9LIQ9_ARATH	Protein BLISTER
	isotig04489	3	3	G7K8N7_MEDTR	EH-domain-containing protein
	isotig04509	3	3	B9GPQ2_POPTR	MLO-like protein
	isotig06416	3	3	B9GXB4_POPTR	Predicted protein
	isotig04542	3	2	B9SN36_RICCO	Protein SEY1 putative
	isotig04555	3	3	Q2HV31_MEDTR	Prefoldin
	isotig04565	3	3	No hit	
	isotig04726	3	3	B9H3Y3_POPTR	Predicted protein
	isotig04609	3	2	G7IAZ5_MEDTR	Protein kinase Pti1
	isotig04623	3	3	B9RUS7_RICCO	Kinesin light chain putative
	isotig04672	3	2	Q5DK69_SOLLC	Pollen-specific kinase partner protein
	isotig05404	3	3	B9H563_POPTR	Predicted protein
	isotig04693	3	3	No hit	
	isotig04767	3	3	CAP4_ARATH	Putative clathrin assembly protein At1g03050
	isotig04789	3	3	B9HB66_POPTR	Phosphatase 2C family protein
	isotig04845	3	3	B9SV82_RICCO	UDP-glucose 4-epimerase putative
	isotig04879	3	3	D7KWK7_ARALL	Kinase
	isotig04904	3	3	B9S7I4_RICCO	Myosin XI putative
	isotig02271	3	3	B9HI16_POPTR	Predicted protein
	isotig04919	3	3	B9RZI2_RICCO	Protein binding protein putative
	isotig02491	3	2	B9HIN8_POPTR	Predicted protein
	isotig05341	3	3	B9HRU2_POPTR	Predicted protein
	isotig05158	3	2	B9SLE6_RICCO	Calcium-dependent protein kinase putative
	isotig05212	3	3	B9RBM2_RICCO	26S proteasome regulatory subunit S3 putative
	isotig05236	3	2	Q9SXT7_CICAR	Rac-type small GTP-binding protein
	isotig05248	3	3	B3TLS7_ELAGV	Senescence-associated protein
	isotig04529	3	3	B9I173_POPTR	Predicted protein
	isotig05448	3	3	A5XEM2_MEDTR	Putative RNA binding protein
	isotig05474	3	3	Q8GWT7_ARATH	Putative embryonic abundant protein
	isotig05541	3	3	Q8L7U7_ARATH	AT4g39670/T19P19 60
	isotig05557	3	3	E0A8M9_PETIN	VAMP721
	isotig05584	3	3	B9RK58_RICCO	Ectonucleotide pyrophosphatase/phosphodiesterase putative
	isotig05652	3	3	B9T3W8_RICCO	Troponin C skeletal muscle putative
	isotig05683	3	2	B9RH93_RICCO	Kinase putative
	isotig05738	3	3	G8A187_MEDTR	40S ribosomal protein SA
	isotig05759	3	1	B9VS69_9ASTR	Heat shock protein 70.58
	isotig05797	3	3	B9SE94_RICCO	C putative

Allergen name	Transcript number	Sequenced peptides	Unique sequenced peptides	Homolog uniprot entry	Annotation
	isotig05807	3	3	H6TNS0_ELAGV	Putative 24 kDa seed maturation protein
	isotig03480	3	3	B9IGR3_POPTR	Predicted protein
	isotig05842	3	3	Q9ZNX3_PETHY	PGPS/D7
	isotig05877	3	3	Q8L8T0_ARATH	Ripening-related protein-like
	isotig07106	3	3	B9IHT1_POPTR	Predicted protein
	isotig05586	3	3	B9ILW2_POPTR	Villin 4 family protein
	isotig06175	3	3	D7TAR8_VITVI	Proteasome subunit beta type
	isotig06266	3	3	D7MI82_ARALL	ATCHX24
	isotig06295	3	2	B9SA27_RICCO	Trafficking protein particle complex subunit putative
	isotig06348	3	3	B9RJX8_RICCO	Restin putative
	isotig05878	3	2	B9MY35_POPTR	Predicted protein
	isotig06521	3	3	B8Y8A0_GOSHI	Blue copper-like protein
	isotig06542	3	1	G7KHL3_MEDTR	ADP-ribosylation factor
	isotig06546	3	3	B6T7E9_MAIZE	Putative uncharacterized protein
	isotig06551	3	3	MSRA_FRAAN	Peptide methionine sulfoxide reductase
	isotig06569	3	3	B9RK06_RICCO	Pre-mRNA-splicing factor cwc24 putative
	isotig06737	3	3	B9SB16_RICCO	Rhicadhesin receptor putative
	isotig06810	3	3	RL10_VITRI	60S ribosomal protein L10
	isotig06854	3	2	Q9M6E6_TOBAC	Poly(A)-binding protein
	isotig06940	3	3	Q96403_CUCSA	Stellacyanin
	isotig07017	3	1	Q6V5G1_9BRAS	Cu2+ plastocyanin-like
	isotig07260	3	1	G7KP54_MEDTR	GPI-anchored protein
	isotig07686	3	3	B6T2I0_MAIZE	60S ribosomal protein L11-1
	isotig07711	3	3	G7LBH9_MEDTR	Protein vip1
	isotig07781	3	3	GPX1_HELAN	Glutathione peroxidase 1
Amb a 10	isotig07261	2	2	Q2KN25_AMBAR	Calcium-binding protein
	contig09392	2	2	WNK8_ARATH	Serine/threonine-protein kinase WNK8
	contig23568	2	2	Q6EP48_ORYSJ	Os02g0579800 protein
	isotig01056	2	1	B9T805_RICCO	Serine/threonine-protein kinase PBS1 putative
	isotig01686	2	2	C0IXK1_SOLME	U2 snRNP auxiliary factor large subunit
	isotig02009	2	2	B9R9X1_RICCO	Acid phosphatase 1 putative
	isotig02303	2	2	G7ILD5_MEDTR	Syntaxin-124
	isotig02327	2	2	B9SSY3_RICCO	Type II inositol 5-phosphatase putative
	isotig07099	2	2	A9PE36_POPTR	Predicted protein
	isotig02542	2	2	Q9M6E6_TOBAC	Poly(A)-binding protein
	isotig02641	2	1	B9RRX5_RICCO	Serine/threonine-protein kinase putative
	isotig02724	2	2	B9HCD3_POPTR	Autoinhibited H+ ATPase
	isotig02815	2	2	D2U833_9ROSI	Cation chloride cotransporter
	isotig03358	2	2	B9GFY6_POPTR	Predicted protein
	isotig02871	2	1	D7SPC8_VITVI	Putative uncharacterized protein
	isotig03136	2	2	Q5I6E8_SOLLC	3-phosphoinositide-dependent protein kinase-1
	isotig03188	2	2	Q5NT80_TOBAC	Potassium channel NKT1
	isotig03227	2	2	G7JCD0_MEDTR	Plasma membrane H+ ATPase
	isotig03250	2	1	B9S7I4_RICCO	Myosin XI putative
	isotig05836	2	2	B9GJS3_POPTR	Predicted protein
	isotig03264	2	2	B9RY53_RICCO	Gcn4-complementing protein putative
	isotig03546	2	1	I1Y996_NICBE	RabE1
	isotig04771	2	1	B9GN25_POPTR	Pleckstrin homology domain-containing family protein
	isotig03552	2	2	G7J8X4_MEDTR	RHO protein GDP dissociation inhibitor

Allergen name	Transcript number	Sequenced peptides	Unique sequenced peptides	Homolog uniprot entry	Annotation
	isotig03580	2	2	HIS8_NICPL	Histidinol-phosphate aminotransferase chloroplastic
	isotig03600	2	2	Q53XI0_ARATH	Calcium-transporting ATPase
	isotig03616	2	2	B9R792_RICCO	Polyadenylate-binding protein putative
	isotig06274	2	2	B9GQW2_POPTR	HVA22-like protein
	isotig03914	2	2	B9GR88_POPTR	Late embryogenesis abundant domain-containing family protein
	isotig04239	2	2	B9RX93_RICCO	Zinc binding dehydrogenase putative
	isotig04279	2	1	D3GC04_9ROSI	Copper chaperone
	isotig04322	2	2	G7L3J8_MEDTR	DEAD-box ATP-dependent RNA helicase
	isotig04423	2	2	Q93XQ3_NICAL	Putative beta-1 3-glucan synthase
	isotig04429	2	2	B9RQ57_RICCO	Ubiquitin carboxyl-terminal hydrolase
	isotig05695	2	2	B9GX91_POPTR	Predicted protein
	isotig04444	2	1	B9SDM2_RICCO	Calcium-dependent protein kinase putative
	isotig04461	2	2	E5GBL8_CUCME	ATP-dependent clp protease
	isotig04500	2	2	Q9LW99_MESCR	Protein kinase MK6
	isotig04507	2	2	Q9LKQ9_HELAN	Asparagine synthetase
	isotig04515	2	2	Q3YAT0_PETIN	Calcium-dependent protein kinase 2
	isotig04539	2	2	G7LI83_MEDTR	Aminophospholipid ATPase
	isotig04577	2	2	B9S6T7_RICCO	Plant ubiquilin putative
	isotig04587	2	1	B9RGR3_RICCO	Serine/threonine protein kinase putative
	isotig04876	2	2	B9H4R0_POPTR	Predicted protein
	isotig04651	2	2	B9GPQ2_POPTR	MLO-like protein
	isotig07167	2	2	B9HEA7_POPTR	Predicted protein
	isotig06146	2	2	B9HFS8_POPTR	Hexokinase
	isotig04906	2	2	F4JZ24_ARATH	Iron ion binding / oxidoreductase/ oxidoreductase protein
	isotig05910	2	2	B9HI74_POPTR	Predicted protein
	isotig04925	2	1	B9HJT8_POPTR	Pectate lyase
	isotig04948	2	2	G7IJV4_MEDTR	Serine/threonine protein phosphatase 6 regulatory subunit
	isotig05011	2	2	B9T214_RICCO	Rho GDP-dissociation inhibitor putative
	isotig06549	2	2	B9HKT2_POPTR	Predicted protein
	isotig02880	2	2	B9HLK9_POPTR	Phosphoglucosamine mutase family protein
	isotig02446	2	2	B9HNR2_POPTR	Predicted protein
	isotig05079	2	2	B9HQE4_POPTR	Predicted protein
	isotig06193	2	2	B9HRF6_POPTR	Kinesin-like protein
	isotig05102	2	2	Q711G8_TOBAC	Cullin 1A
	isotig05103	2	2	NDK_HELAN	Nucleoside diphosphate kinase
	isotig05132	2	2	G7JW19_MEDTR	Vesicle-associated membrane protein
	isotig05138	2	2	B9T2I4_RICCO	Acyl-CoA thioesterase putative
	isotig05045	2	2	B9HTY1_POPTR	Predicted protein
	isotig05357	2	2	B9HZX5_POPTR	WD-40 repeat family protein
	isotig05299	2	2	F2DLW6_HORVD	Elongation factor 1-alpha
	isotig05370	2	2	B1PVT0_TOBAC	LIM domain protein 2b
	isotig05377	2	2	Q41152_RICCO	Sucrose carrier
	isotig05443	2	2	B9RUL1_RICCO	Acid phosphatase putative
	isotig05452	2	2	Q94CF8_CAPAN	Branched-chain-amino-acid aminotransferase
	isotig05320	2	1	B9I763_POPTR	DEAD box protein P68
	isotig05519	2	2	G7L857_MEDTR	Galactose oxidase
	isotig05537	2	2	VATL_KALDA	V-type proton ATPase 16 kDa proteolipid subunit
	isotig05566	2	2	B9SDN2_RICCO	Calcium-dependent protein kinase putative
	isotig05578	2	2	Q15FD2_STRAF	NOX1

Allergen name	Transcript number	Sequenced peptides	Unique sequenced peptides	Homolog uniprot entry	Annotation
	isotig05627	2	2	B1PVT0_TOBAC	LIM domain protein 2b
	isotig05696	2	2	SF3_HELAN	Pollen-specific protein SF3
	isotig05699	2	2	B3TM00_ELAGV	Translational initiation factor eIF1
	isotig05718	2	2	B9S0D5_RICCO	Ankyrin repeat-containing protein putative
	isotig05773	2	2	F6H2F7_VITVI	Ubiquitin carboxyl-terminal hydrolase
	isotig05777	2	2	GPX4_HELAN	Probable phospholipid hydroperoxide glutathione peroxidase
	isotig05830	2	2	G7IAA3_MEDTR	Isocitrate dehydrogenase (NAD+)
	isotig05891	2	2	B9SAD4_RICCO	Nuclear acid binding protein putative
	isotig06117	2	2	Q68GS0_TOBAC	SKP1
	isotig06120	2	2	B9RDD0_RICCO	Plant sec1 putative
	isotig06145	2	2	Q9AT32_DAUCA	Poly(A)-binding protein
	isotig06164	2	2	B9RM00_RICCO	Beta-hexosaminidase putative
	isotig06208	2	2	B9RDP2_RICCO	Dead box ATP-dependent RNA helicase putative
	isotig06211	2	2	Q9AVP0_TOBAC	Ubiquitin-conjugating enzyme (E2)
	isotig03070	2	2	B9MVE1_POPTR	Predicted protein
	isotig06393	2	2	B9RGI7_RICCO	Oligopeptidase B putative
	isotig05234	2	2	B9MZ16_POPTR	Pantothenate kinase-related family protein
	isotig06532	2	2	G7LH86_MEDTR	Phi-1 protein
	isotig06624	2	1	IF5A_SENVE	Eukaryotic translation initiation factor 5A
	isotig06655	2	2	B9RG74_RICCO	NADH dehydrogenase putative
	isotig06782	2	2	B9SRJ0_RICCO	Chemocyanin putative
	isotig06799	2	2	CYT5_ARATH	Cysteine proteinase inhibitor 5
	isotig06858	2	2	No hit	
	isotig06949	2	2	No hit	
	isotig06950	2	2	B9SZE8_RICCO	Catalytic putative
	isotig06969	2	2	No hit	
	isotig07044	2	2	No hit	
	isotig07116	2	2	B9SDM9_RICCO	Sucrose phosphate phosphatase putative
	isotig07215	2	2	B9RC95_RICCO	Actin depolymerizing factor putative
	isotig07244	2	2	No Hit	
	isotig07301	2	2	B9GJS2_POPTR	Uridine kinase
	isotig07445	2	2	PSA4_SPIOL	Proteasome subunit alpha type-4
	isotig07523	2	2	B9RM96_RICCO	Protein binding protein putative
	isotig07535	2	2	B6UH40_MAIZE	WNK6
	isotig07688	2	2	Q8LAM9_ARATH	Pollen coat-like protein
