# Supplementary material for: Identification of Novel Short Ragweed Pollen Allergens Using Combined Transcriptomic and Immunoproteomic Approaches
Source: PLoS One. 2015 Aug 28;10(8):e0136258. doi: 10.1371/journal.pone.0136258 (PMC4552831; doi:10.1371/journal.pone.0136258)
Supplement: S2 Table — provides the nucleotide sequences, recovered from the transcriptome analysis, encoding the identified candidate allergens. (PDF) [file pone.0136258.s003.pdf]

S2 Table: nucleotide sequences of candidate ragweed pollen allergens

S2 Table provides the nucleotide sequences, recovered from the transcriptome analysis, encoding the identified candidate allergens

Name	Nucleotide sequence
Amb a 1-like	ATGTTTAATCTCTTGGTGCAAATTATTTGACGCACATTATTAGTTCTTGGTAAACCGATAATTACTGAATGTATATAAGCAAA<br>AAGTTGTTAAATAAATGATGAGATCGATCACCAAGAAGAACCACAACAATCATACAATGGGGATCAAACACTGTTGTTACAT<br>CTTGTATTTTACCTTAGCCCTTGTCACTTTGCTGCAACCTGTTCGTTCTGCCGAAGATCTCCATGAAATCTTACCTTCACCTA<br>ACGGAACAAGGAGGGGCCTGCAAGCATGTGAAGCAAACAACATTGTAGACCAGTGCTGGAGGTGCAAAGCGGATTGGGC<br>GGAAAACCGACAAGCGTTAGCCGATTGTGCCCCAGGTTTTGCAAAGGGAACCTTCGGCGGAAAATTGGGTGATGTCTACA<br>CCGTGACAAACGAAAATGATGATGATGTTGCAAATCCAATAGAAGGGTCACTCCGGTTTGGTGCGGCCCTGGAAAGGCCC<br>ATATGGATCATTTTTGAAAGAGATATGGTGATTCATTTGAACCAAGAGCTTGTGATAAACACAGACACGACGATCGATGGG<br>CGAGGGGCGAATGTAGAGATCATTAACGCTGGTTTGCACATCAATAATGTCAAGAATGTAATCATTCATAACATAAATATAC<br>ATGATATTAAACCGAATGAGGGAGGCATTGTTAAGTACAACGATGGTCCAGCAAGAAAAAGACCCAAAAGTGATGGTGATG<br>CTATATATATTGCTCGTGCTTCACAAGTATGGATCGACCATTGCTCGCTCAGTATGACTGCCGATGAGCTGATCGATGTCA<br>AACTCGGCAGCACGATGGTGACCATTTCCAACTGCAAATTCAGCCAAAGCCAATTTGTATTATTGTTGGGGGCTAATGACG<br>AAGATGTTGAAGATAAAGCCATGATGGTAAATGTGGCATTCAACACGTTCGCCGATCAGGTTGACCAAAGAATGCCCAGAT<br>GTCGACATGGGTTTTTCCAAGTCGTTAACAACAACTACGACAGATGGGGAACGTACGCTCTCAGTGGTAGCGCGGCTCCA<br>ACTATACTCAGCCAGGGGAACAGATTCTTCGCCGGGGATGAGCCCTCCAAGAAAAGTGTTGTAATGAGGAGTGGTCCAGG<br>CACCGAAGGGTCGATGTCGTGGAACTGGAGATCGGATAGAGACTTGCTTGAAAATGGTGCTACTTTTGAACCATCTGGGG<br>TTGATCCAGTGCTAACCCCTGAGCAAAACGCAGGGATGATTCCTGCCGAACCTGGAGAAGCCGCTCTAACACTCACTGCT<br>TTTACTGGTGTACTCTCATGCCAGCCTGGAGTACCTTGCTAAGCACCCGACCAATTTTATAATGTTCATAAAGACGTTTTTT<br>GTGTTGTTTTTTTAGTTAATAATATGCGACGATAAATTATACAATCTAAATCTATGTTAATCATGAATTTAGATTATCAATTAAT<br>GTTCATAAAGTGTATCGGCTACACCGATGGATGTTTCAAAACTTTATGTGAGGAAGAAGGAAGTAGTTTCAATTATAATATA<br>CTAGTATGAATATGAATGTTTGTGCGTTACC

Name	Nucleotide sequence
Carbonic anhydrase	AAAAATTACCTTAAATGAAACACAAACCTCAACCATGAATTTCTCCAAACCGCTAATTAAATCATTAACCAATAAACAATTTAAAGCTAAATGTGAAAACGATAAGCCACACAGATTTTGAATCTAGCTTGTCCAACAATGTTTACATGATTACATACATAGTCACGATTCAAGAAAAACTCGAACCATCTCTTCAATTCCATAACGATGTCAAACCTTAACCTATCATCACTTCTATTCATTTGTCTTATGATCATATCATCACGCTCCTTCATTTCCATTTGCACGGCTAATGAGTGCAATGATGTAGGGTTACGTCAACAACTCTTGTTTGGAACAGCAACGAATGACATGCCCCAAACCACCTTTGATCGTCGAAAAATCTTGGAGATTGCAGAAGGTGGGCCAAAATTCAACTATCTAGAAGGGACTCAACTTGGTCCAAAGAATTGGGGAACTATTACGCCGGAATGGAAAATTTGTAACGATGGGAAATCACAGTCTCCTATTAATATCGAAAACAATAGGGTGCAAGTTACGCCAAGTGACTTGAAACGTGCTTACCTGGACGCGCCAGCCACAATTATTAATGGAATTAATAATATTGGTGTTCGTTGGACAGGAGATGGGGGAGGAATAGAAGTAAATGGTTCAATATACAAGCTAACTCAATGCCATTGGCATACTCCTTCTGAGCACACTGTTGACGGGAAGGGGTTCGATGCAGAACTACACCTGGTTCATAAAAATGCCATGGGGCGTTTGGCGGTTGTTGGCATGCTTTACAACATTGGACTGCCTGATCCTTTTATTACAAGTCTAGCACCGATGATGAGTGTACTTAATATAAATGAAGAAATTGCTGTGCCGAAAATAATTGCAGCTAGTATAAACTTTGGCAGCAAAAAGAACTTTAGATATTATGGTTCCCTTACTACTCCTCCTTGCTCGGAGAATGTTATTTGGACTGTTGAAGAAAGGGTTCGAACAATTTCACGCGAACAGATCAACCTGCTTAAGGGAAGTCATCAACCGGAATATCAACAAAATGCAAGGCCAGTACAACTACTTGGAGACAGACAAATTTGGAGTTTTGATTTACCTGAACCAGTCGTGTAGACTCGACTTATTCATGAAAGGCGTATTTATTTATTTATTTACATTTGAAAACATAAAATAATAAATCATTTTATGTCTGTATAGTATAATTTAAAAGGATATTAAATCATATTGTCAGCATATTTTTATATGCTATTAATTAATAACGATCT
Enolase	TAAAGCTGGATACACAGACAAGGTTGTCATTGGAATGGATGTTGCCGCATCTGAATTCTATGGAAAAGGACAAGACCTATGATCTGAACTTCAAGGAAGAGAACAACGATGGCAAAGAAAAGATTTCAGGAGAACAACTAAAAGATCTTTACAAGTCGTTTGTGAGTGAGTACCCCATTGTGTCCATTGAGGACCCATTTGACCAAGATGACTGGGAGCACTATGCCAAGATGACCGCTGAATGTGGCGAACAAGTACAAATTGTAGGAGACGATCTTTTGGTCACCAACCCACGAGAGTCAAGAAGGCAATTGATGAGAAGACTTGCAATGCTCTTCTTCTAAAGGTTAACCAAATTGGGTCTGTGACCGAGAGTATCGAAGCTGTGAGGATGTCCAAACATGCTGGTTGGGGTGTGATGGCCAGTCACCGCAGTGGAGAAACAGAGGACACCTTCATTGCTGATCTTTCTGTGGGTTTGGCAACGGGTCAAATCAAAACTGGAGCTCCATGCAGGTCAGAGCGTCTTGCAAAGTACAACCAGCTGTTGAGAATCGAAGAAGAGCTTGGATCAGAAGCGGTTTATGCTGGAGCCAACTTCCGCAAGCCAGTGGAACCCTACTAGATTTTACACAGATGACGATGAGTCGAGATGAGTGTCCATGTGGAATACGGCAATCACGTAGACGTGATGTTAATAAGGGCTACTCGAGTTTTATTTTGAGGAGGTTTTTAACTTCTAAAATTTGCAGTTTGG

Name	Nucleotide sequence
GDP dissociation inhibitor	GGTCCAGAAAAACAGCAGTCCATTCAAACTTTCCATTTCTATGCCTTCTCTTCCTCTATAATTTGTGGAACACACATTACATT ACGTTATCAACCTCTTTAATTTGTGTTTCTTATTCTACAATCTCCATTTCTTCATCATCTTCTTTTTTATATTATTTGTTGAATAT ATCAGTGAGATAATTGTTTGTGTAGATCATATCAACTATGGATGAAGAATACGACGTCATAGTTCTTGGCACCGGCCTCAAA GAATGCATCCTCAGCGGTCTCCTCTCCGTCGATGGCCTCAAAGTGTTGCATATGGATAGAAACGATTATTATGGAGGAGAA TCGAGCTCTCTCATGTTAAATCAGCTTTGGAAGCGGTTTAAAGGAGATGAAAACCCTCCGGAAACTTTAGGACTAGCAAGG AGTATAATGTTGATATGATTCCAAAGTTCATGATGGCAAATGGTTTGTTGGTTCGCGCTCTGATCCACACCAACGTTACAAA GTATCTGAATTTCAAGGCGGTGGATGGTAGCTTTGTATACAGCAATGGGAAGATCTACAAAGTTCCTGCAACTGACGTTGA AGCGCTGAAATCTTCGATTATGGGCCTGTTTGAAAAAAGGCGCGCACGAAAGTTCTTTATATATGTTCAAGATTTTGAGGAA AACGATCCCAAATCTCATGAAGGTTTGGATTTGAACACAATTCCTGCAAAAGAGCTCATTAGTAAATATGGTCTTGAAGATG ATACAATTGAATTTATCGGTCATGCATTGGCACTCAATACTAGTGACAGTTATTTGGAACAGCCCGCAATCGATTTTGTGAA GAGAATCAGGCTATATGCAGAGTCCTTAGCACGTTTCGAATCAGGATCTCCTTACATTTACCCAATGTATGGGCTTGGAGA GTTGCCACAGGCGTTTGCTCGATTAAGTGCAGTTTATGGGGGGACTTACATGCTTAACAAGCCACAATGTAAGATAGAATT TGATGACAGTGGGAAGGTGATTGGTGTGACCTCTGATGGAGAAACTGCTAAATGCAAAAAAGTTGTATGTGATCCATCATA CGCGCCCGATAAGGTCAAAAAAGTTGGGAAAGTTGCTCGAGCCGTATGTATAATGAGTCATCCGATTCCAGATACAAACGA TTCTCACTCGGCCCAAGTCATTATTCCACAGAAGCAACTTAACCGTAAATCAGACATGTATCTATTTTGCTGTTCTTATTCAC ACAGTGTGGCTCCACAAGGCAAGTTTATTTCATTTGTGACAACGGAAGCAGAGACTGACGACCCTGAGAGCGAATTAAAG CCCGGCGTTGACCTTTTGGGACCCGTGGATGAGATCTTTTATGAAACTTTGGACAGATTTGAGCCAACAGATGATGGTGCT GCTGATTGCTGCTTTGTATCTACTAGTTATGATGCAACGACACACTTTGAGACAACGATGATTGATGTTCTTAACATGTACA CCAAAATAACCGGAAAGGAACTTGACCTTTCGTGGACCTGAGTGCCGCTAGTGCTGGTGGTACCGAAGAATAAGTCGTTT GCATCAAAACCTCTTGGCTTGTGGAGAGCTTTATTTCACACAGTCGGATAACGGAATATATTCTTTATTGTTTTGAAAATAG GAATTGATGATCTGCACATCATGCTATTTTCTCATTTTAATATCACAAAGAAATAATTGTTCGAGTTTGTTTTGTAATAATTAC TTGATAATTGATCCCCAAGTCCCAAGCTGTACCCCT

Name	Nucleotide sequence
Polygalacturonase #1	GGTGAATCCACGGAAATTCAGAAACACCAAGAAATCGGGTGTTTCATAAAAATTTTGTTCTAATTTTAGAATGTCTTTAACAT CATAAATAGTAAAACCATCAAACACATCTTTTGCATATCTAACCATTCAATACAAACCCAAAAATCTAGTCACAATGGAACTC AAATCATGTCTAATTTGCGCCATTTTTATGTTCATAGCCATCGCGCAGGCTGAAGTTTTCAACATCATGGATTTCGGTGCAA AGCCGGATGAGGAGATTAGCACGGCTCTTAAAGATGCATGGGCCAAGGCATGTGCTGCAACCGAACCGAGCCAGCTGAT CATTCCAACAGGCCGATTTCGCTTGAACGTGATCGAGTTTAAGGGACAATGTAAGGCACCAGTCGAGGTTAAAATCGATGG AACTGTCATAGGTCCAGAAGATCCTATGGCCATTCCAAAAGGTGTCCAATGGATCACATTTAGCTACGTAACAGGCTTGAC ATTATCAGGTTCGGGTACGGTAGATGGTGTTGGAGCAGCCATGGCATGGCCATCAAACAACCAGAAAATAACAAGAAAAG GCGCATGCGGCCTTCAATATAATCTTTGCTTTAACTTCATTAACGATTCTATGGTTACTGGAATAACTTCAAAAGATAGCAAA AACTTCCACGTGAATTTGATGGCATGTACGAATCTTACATTTGATAATTTCCACATAAGCGCACCCGCAGAGAGCCCCAAC ACGGATGGAATCCACATCGGATGGTCGAAAAACATCACAATCATGAATTCCATTATTGAAACGGGTGATGATTGTGTGTCG ATTGGCGATGGTTGTGAAAACTTGAACATCGTGGGAACGAAATGTGGTCCGGGCCATGGAATTAGCATTGGAAGTTTGGG TAGAAACAAGGGTGAGAAGCCCGTGATCGGGGTTTACGTGAAGAACTGCTCGTTTTTAGAGACGGACAACGGTGTTAGGA TCAAAACATGGCCTGATTCGCTGCCCGGAGAAGTTACCGAAATGCATTTTGAAGATCTTATCATGGATAAAGTTAGGAACC CTGTTATCATCGATCAAGAATATTGCCCTAATAACGATTGCAACAAAGATAAACCATCGATGGTTAAGGTTCACAATGTTTTC ATCAAGAATGTTGCTGGAACTTCGAATACACCTGAAGTTGTGAAGCTTATTTGTAGCAAGGCTAACAATGGATGTGAGAAT GTGCACGTTTCCGACATTAATTTGACATATGATGGTCCTGAAGGCCAAGCATTTCAAGAGTGCCGTAATGTGAAGCCGATT TATGAGGGCCAACTTAACCCTCCTGGCTGTGCAGCGGCTTGATCCTATTGAAAAGGGATTCAAACTTATGCATGGTTGCAT GCAGTCGTTGCTGGTTCTCCACAAAAGAAGGCTGGTCTTTTGTTATCATTTTGAATTGCTTTTGAATAAGTTGTTACGTCAA GTTGTTGAGTCTTCCTGTTTTTCTGGTATTAGCCGTTTGATAATTTAGTATAAGTAGTAGGCATTGCTTTGTATTCTGTATTC ACTTTCATTTTCATTCAATAAGAGAACCAAGCATTTGAGTCTT

Name	Nucleotide sequence
Polygalacturonase #2	GATATCTAATCATTCAATTCAAACCCAAAAATCTAGTCACAATGGAATTCAAATCATGTCTAATTTGCGTCATTTTTATGCTCATTGCCATCACGCAGGCTGAAGTTTTCGACATCACGAAATTCGGTGCTAAGCCCGATGGAGAGATTAGTAAGGCCTTGTATGAGGCATGGGTCGCTGCATGTTCAGCAACTCAACCGAGCCAACTGCTCATTCCCAAAGGCCGATTTTGCTTGAATGTAATCGAATTTAACGGACCATGCAAATCTCAAGTTGAGGTTAAAATAGATGGAACTTTGGTTGCTCCTGAAGATCCTGTAGTCATTCCAAAAGGATCCCAATGGATCACATTTAGCTATGTCACTGGCTTGAAAGTAACAGGTTCGGGTACGTTAGATGGTGTTGGAGGGGCCATGGCATGGGTAACGGACGACCCGAAAACAACTGTCGAAAAAGGCTCAACCCTTCAACATAATCTTAGCTTTAACTTTATCAAAGATTCTTCGATTACCGGAATAACTTCAAAGGATAGCAAGAATATCCACGCGAATATGGTGCAATGTACGAATGTTACATTCGATAATTTCCATGTCAGTGCGCCTGGAAAGAGCCCCAACACGGTTGGAATCCAAATAGGGTGGTCGAAAAACATCATTATCAAGAATTCTGTTATAGAAACTGGCGACGATTGTATCTCAGTTCGCGATGGAAACCTAGATGTGCACATTGAGGGAGTGAAATGTGGCCCGGGCCGTGGTATTAGCATTGGAGGTTTGGGTAGAAACAATGGTGAGAAACCCGTGGCCGGAGTTTTCGTGAAGAACTGCTCGTTTTCGTCCACAGCAAACGGTGTTTGGATTAAAACATGGCCCCATTCACATCCTTTAGATGTTTCCAATATTCATTTTGAAGATCTTACCATGGATAAAGTTGGCAACCCTATTTTCATTCAGCAAGATTATTGCCCACATATCGAATGCAAAAGAGATAGTCCATCAATGGTTAAGATTCATGATGTCTTCATCAAGAATGTTACGGGAACTGCGAATTCGCCTGAAGTTGTGAAGCTGAGATGCAGCAAGGCCAGTAAAGGATGTGAGAATATTCAGATTTCAGACATTAACTTGAAATATGATGGTCCTGCAGGCAAAGCAATTCAAGAATGCCAACACGTGAAGCCGGTTTATAGCGGTCAACTTATCCCTCCAGGTTGTGCAAAGGCTTTTGAAATGTAACTCAATGTTAAAGATAGCCGTTTGCGTTAGAATAAGAGTGGGTAAGTATGAGTCCTGGCTTTAGGAATTAGGATAGCTTGTTGCACAATAAACGTGTGGTCTTTTATGTATGTTTTTGTTTAGATTTAAAGTCGTTTGGTTGCTTGTTTAAAGGCTTTTATTGATCAATAATACATGCAC
PR-17	GCGGGCATGATTAAATTCAACACAGTTATTGGAGGAGTTCCATACACAAAACAAATAATGGGTGAGATCAACAAAATGTTATGGTCAGTGTTTAAACAAAACACCCCGGCTGAGCGAAGACCTGTAGATACCGTTCGCGTATTACTAAATGATAATACAGGACCGGGCATAGGAGTCACTTTGGGAGACACGATTAATATTAGCATGAGTTTTATAGGCAAGTACACTGGGCCAGTGGAATTAAAAGTGGTATTCGCGGCCCTTTTGCACCATGAAATGGCGCATGTTTTCCAATGGTTTGGCGGGGGTAAGACCCCTCAAAATTTGATAGAAGGGATCGCAGAGTACACAGTTATCAAAGCTAATTATAAAGGAATAATCTTTGAGAAGCCTGGTTCTGGGGATAAATGGGACAAGGGATATGCTTTCACAGCCCGTTTTCTCGAGTATTGTGATGGGCTCGTTCCAGGGTTCGTGGCGCGACTTAATAATATGATGAGGAACAATTACGATGTCTCGTTTTTTAAAAGTATCACAGGAAAGCCTGTGGAGCAATTGTGGAAGGAATACAAGGCTAAATATCCGGGGGT

Name	Nucleotide sequence
UDP-glucose pyrophosphorylase	ATTCATTCATTTTATTTTTCTTATCATCATCATCATCATCATAATATCATTACTCTAGATCCAAATCAATCAATCAATCAATGGC<br>CGCTGCTGATACCGAGAAGCTGAACAATCTCCGATCTGCTGTCTCCTCTCTCACTCAGATCAGCGAGAATGAGAAATCTGG<br>ATTTATCAACCTTGTGTCCCGCTATCTCAGTGGCGAAGCAGAACATGTTGAATGGAGCAAGATCCAAACGCCTACTGACAA<br>GATCGTTGTGCCCTATGATACCCTATCAGCTGTACCAGAAGATGCTGCTGAAACCAAAAGTCTATTGGATAAGCTTGTGGT<br>GCTAAAGCTTAATGGTGGCTTGGGGACAACAATGGGATGCACTGGTCCAAAATCTGTCATAGAAGTGCGAAATGGATTGA<br>CATTTTTAGACTTGATTGTCATCCAAATTGAGTCACTCAATAAGAAGTATGGCTGTAGTGTACCCTTGCTTCTAATGAACTCA<br>TTCAACACACATGAAGATACCCAGAAGATTATTGAAAAATATGCTGGTTCAAATATCGAGATTCATACATTCAATCAGAGTCA<br>ATATCCTCGATTGGTTGTTGATGACTTTCTGCCACTGCCATCTAAAGGGGAAACCGGCAAAGATGGATGGTACCCTCCAGG<br>GCATGGTGATGTTTTCCCATCCTTGATGAACAGTGGGAAACTCGATGCACTACTGTCTCAGGGCAAGGAATACGTCTTTGT<br>TGCAAATTCCGATAACTTGGGAGCTGTAGTTGATTTGAAAATCTTGAATCACTTGATCCAGAACAAGAATGAGTACTGCATG<br>GAGGTAACACCTAAAACATTGGCTGATGTTAAAGGTGGCACTCTAATTTCATATGATGGGAAAGTTCAGCTTCTTGAAATTG<br>CACAAGTTCCCGATGAGCATGTCAATGAGTTCAAATCAATTGAGAAGTTCAAAATCTTCAACACCAACAACTTGTGGGTGAA<br>CTTGAATGCAATTAAAAGACTTGTGCAAGCCGATGCACTTAAGATGGAGATTATTCCAAATCCAAAGGAAGTCAATGGAGTT<br>AAAGTTCTTCAGCTTGAGACAGCTGCTGGTGCTGCAATCAAGTTTTTTGACAATGCCATTGGCATTAATGTTCCCCGATCTC<br>GATTCTTGCCGGTGAAAGCAAGTTCAGATTTGCTTCTTGTTCAGTCCGATCTTTACACTGAAAAAGATGGCTATGTGATCCG<br>CAACCCAGCTAGGACAGATCCAGCTAATCCTTCAATTGAATTGGGTCCTGAATTTAAGAAGGTTGGAGATTTCTTGAAGAG<br>GTTCAAGTCTATCCCCAGCATCATTGAGCTTGCTAGCTTGAAGGTTTCTGGTGATGTGTGGTTTGGATCTAATGTTGTTCTC<br>AAGGGTAAAGTGGTGGTTGCTGCAAATTCTGGAGAGAAGTTGGAAATTCCAGATGGAGCTGTACTTGAAAACAAGGAAGT<br>GCACAGTGCTGGTGATATCTAAAAGGTTATGGTTTGGTGTGTGGCAAGAGGTTACTTCCAAGTTGGTAATAATAATAATGTG<br>TTGTTTAGTATTTTTAGAATAATAT
